# Functional Characterization of the *Arabidopsis* Ammonium Transporter AtAMT1;3 With the Emphasis on Structural Determinants of Substrate Binding and Permeation Properties

**DOI:** 10.3389/fpls.2020.00571

**Published:** 2020-05-21

**Authors:** Dong-Li Hao, Shun-Ying Yang, Shu-Xia Liu, Jin-Yan Zhou, Ya-Nan Huang, Anne-Aliénor Véry, Hervé Sentenac, Yan-Hua Su

**Affiliations:** ^1^State Key Laboratory of Soil and Sustainable Agriculture, Institute of Soil Science, Chinese Academy of Sciences, Nanjing, China; ^2^Department of Computational Biology, Beijing Computing Center, Beijing, China; ^3^BPMP, Univ Montpellier, CNRS, INRAE, Institut Agro, Montpellier, France

**Keywords:** AtAMT1;3, electrophysiological assessment, point mutation, substrate binding, transport mechanism, homology modeling

## Abstract

AtAMT1;3 is a major contributor to high-affinity ammonium uptake in *Arabidopsis* roots. Using a stable electrophysiological recording strategy, we demonstrate in *Xenopus laevis* oocytes that AtAMT1;3 functions as a typical high-affinity NH_4_^+^ uniporter independent of protons and Ca^2+^. The findings that AtAMT1;3 transports methylammonium (MeA^+^, a chemical analog of NH_4_^+^) with extremely low affinity (*K*_m_ in the range of 2.9–6.1 mM) led to investigate the mechanisms underlying substrate binding. Homologous modeling and substrate docking analyses predicted that the deduced substrate binding motif of AtAMT1;3 facilitates the binding of NH_4_^+^ ions but loosely accommodates the binding of MeA^+^ to a more superficial location of the permeation pathway. Amongst point mutations tested based on this analysis, P181A resulted in both significantly increased current amplitudes and substrate binding affinity, whereas F178I led to opposite effects. Thus these 2 residues, which flank W179, a major structural component of the binding site, are also important determinants of AtAMT1;3 transport capacity by being involved in substrate binding. The Q365K mutation neighboring the histidine residue H378, which confines the substrate permeation tunnel, affected only the current amplitudes but not the binding affinities, providing evidence that Q365 mainly controls the substrate diffusion rate within the permeation pathway.

## Introduction

NH_4_^+^ and NO_3_^–^ ions are the major nitrogen forms available for the absorption by plant roots. Under physiological conditions, the uptake rate of NH_4_^+^ can be 20 folds higher than that of NO_3_^–^ in *Arabidopsis*, suggesting that NH_4_^+^ can be the major nitrogen source allowing plant growth in this species (Gazzarrini et al., 1999). *Arabidopsis* displays increased biomass production with increased NH_4_^+^ concentrations supplied to the roots, and this stimulation reaches a plateau at approximately 1 mM NH_4_^+^ (Yuan et al., 2007). It is worth to note that this concentration corresponds also to the one at which high-affinity NH_4_^+^ uptake saturates in *Arabidopsis* roots (Loqué et al., 2006; Yuan et al., 2007).

It has been shown that high-affinity NH_4_^+^ uptake in plants is specifically mediated by ammonium transporting proteins (von Wirén and Merrick, 2004; Ludewig et al., 2007). In *Arabidopsis*, the NH_4_^+^ transporter family named AMT contains 6 members. All of them, except AtAMT1;4, are expressed in the roots (Yuan et al., 2007; Yuan et al., 2009), where they play major roles in NH_4_^+^ absorption from the soil. Analysis of knock-out mutant plants has demonstrated that AtAMT1;1, AtAMT1;2 and AtAMT1;3 can mediate together up to 90% of “high affinity” (below 1 mM) NH_4_^+^ uptake activity in *Arabidopsis* roots (Yuan et al., 2007), AtAMT1;1 and AtAMT1;3 respectively contributing to about 30–35% and AtAMT1;2 to 18–26% (Loqué et al., 2006; Yuan et al., 2007). AtAMT1;5 has been suggested to be responsible for the remaining (∼10%) of NH_4_^+^ uptake activity (Yuan et al., 2007). These four AMTs are thought to be effectively coordinated according to their substrate affinities and their spatial localization along the root (Yuan et al., 2007).

In parallel to *in planta* investigations of their physiological roles in NH_4_^+^ transport, mainly by using mutant plants, mechanistic and functional analyses in heterologous expression systems such as yeast and *Xenopus* oocytes can provide information leading to more insightful understanding of the transporting mechanisms and regulation of these systems. The yeast expression system that has been used takes advantage of mutant strains lacking the high-affinity NH_4_^+^ uptake systems. The activity and the overall kinetics of a given foreign AMT can then be determined by functional complementation and labeled isotope uptake experiments (Marini et al., 1997). The oocyte system benefits from the possibility of direct onsite and dynamic observation by high-sensitivity electrophysiological methodology, and is thereby particularly effective in deciphering the transport mechanisms of AMTs. This approach however is restricted to AMT systems mediating electrogenic transport activity and also requires highly stable methodologies for successful recordings of relatively tiny currents. Functional analyses in these heterologous expression systems identified four types of transport mechanisms amongst plant AMTs: (i) NH_4_^+^ uniport (Ludewig et al., 2002, 2003; Wood et al., 2006; Loqué et al., 2009; Yang S. et al., 2015), (ii) NH_3_/H^+^ symport (Søgaard et al., 2009; Neuhäuser and Ludewig, 2014), (iii) NH_4_^+^/H^+^ symport (Ortiz-Ramirez et al., 2011) and (iv) NH_3_ transport (Guether et al., 2009; Neuhäuser et al., 2009).

Such differences in transport mechanisms can be expected to involve specific structural features, as it has been elucidated by numerous structure-function relationship studies with a variety of AMTs from bacteria, fungi, algae and plants (Khademi et al., 2004; Søgaard et al., 2009; Ortiz-Ramirez et al., 2011; Neuhäuser and Ludewig, 2014). The bacterial EcAmtB is the first AMT protein whose crystal structure has been reported (Khademi et al., 2004; Zheng et al., 2004). A deduced model of the central substrate permeation pathway has been used to describe the transport mechanism in EcAmtB (Khademi et al., 2004; Knepper and Agre, 2004), leading to a model that distinguishes three successive steps. (i) Firstly, at the base of the periplasmic vestibule, NH_4_^+^ ions bind to a substrate binding site named S1 (or Am1) by a hydrogen bond with Ser^219^ and by π-bonds with Trp^148^ and Phe^107^ (Khademi et al., 2004; Knepper and Agre, 2004; Zheng et al., 2004). With an essential contribution of Phe^215^, NH_4_^+^ is then deprotonated to the neutral form, NH_3_, which is permeant through the hydrophobic transporter pore (Javelle et al., 2008). However, mutation studies on these residues indicates that F107, despite being part of the NH_4_^+^ binding site, is not essential to conduction of the chemical analog of NH_4_^+^, methylammonium (MeA^+^), whereas F215 is absolutely required (Javelle et al., 2008). In this respect, the precise mechanism of substrate binding to the S1 site is still disputative. (ii) Next, midway in the NH_3_ permeation pathway, the central channel integrates into the membrane with a depth over 20 Å. The width of the hydrophobic pore is confined there by two pore-lining residues, His^168^ and His^318^ (it may also include the contribution of the Leu^208^ on the opposite face). Three NH_3_ molecules are accommodated in the pore and stabilized by the two histidines through hydrogen bonds. (iii) Finally, in the inner vestibule, the NH_3_ molecules return to equilibrium as NH_4_^+^, a phenomenon that is thought to involve the contribution of Phe^31^ (Yang et al., 2007).

Along the permeation pathway, amino acids stabilizing the S1 (Am1) binding site (or “gate” for substrate passage) and the two “pore-confining” histidines (stabilizing the Am2, Am3 and Am4 sites; see Knepper and Agre, 2004) are strictly conserved in AMT proteins, which provides further evidence of their crucial roles in determining the transport activity of AMTs. However, mutations of the first pore-confining histidine in AMTs cloned from different species have been shown to result in opposite (positive/negative) effects on transport activity (Javelle et al., 2006; Boeckstaens et al., 2008; Ortiz-Ramirez et al., 2011; Hao et al., 2016), suggesting that interactions with neighboring amino acids may also contribute to the stability of the conducting pore conformation. This notion is partly supported by other mutations that do not belong to these passage key residues, which have also been reported to tune the activity of plant AMTs (Mayer et al., 2006; Loqué et al., 2009).

In general, the transport of methylammonium through AMTs is considered to involve the same mechanisms as that of ammonium. However, the observation that several point mutations in AMTs result in an opposite effect on the transport activity of NH_4_^+^ and MeA^+^ (Loqué et al., 2009; Ortiz-Ramirez et al., 2011), may indicate that structural divergences exist between AMT proteins in recognizing and transporting the two highly cognate substrates. Actually, despite the extremely conserved key amino acid residues in the substrate binding (S1) and the pore-lining motifs, the overall sequence homologies between plant AMTs and the bacterial EcAmtB protein are generally below 30%, raising the possibility that plant AMTs may function in a rather distinct way from that of EcAmtB and that distinctive and more complex contributions to the substrate binding and passage across the permeation vestibule, resulting from specific structural features, may exist in plant AMTs. It can however be expected that homologous modeling against EcAmtB is likely to be greatly helpful in identifying structural determinants contributing to the functional divergence observed between plant AMT.

Among the three transporters that provide the major contribution to NH_4_^+^ uptake in *Arabidopsis* roots, two of them, AtAMT1;1 and AtAMT1;2, have been successfully characterized by both genetic and electrophysiological approaches. The AtAMT1;1 and AtAMT1;2 transporters displayed distinct substrate transport mechanisms and affinities (Wood et al., 2006; Neuhäuser et al., 2007; Loqué et al., 2009; Neuhäuser and Ludewig, 2014), which can be expected to be physiologically significant by being associated to a spatial coordination among the AMTs in *Arabidopsis* roots (Yuan et al., 2007). Within the framework of this hypothesis, it is a pity that, despite AtAMT1;3 is known to be one of the three major contributors to root NH_4_^+^ uptake, detailed information on its functional properties, in particular the mechanisms of substrate binding and transport, is still lacking.

The present study has aimed at characterizing the functional properties of AtAMT1;3 using a stable electrophysiological recording strategy in *Xenopus* oocytes and at gaining information about the structural determinants of substrate binding and permeation.

## Materials and Methods

### Plasmids Construction

The open reading frame (ORF) of AtAMT1;3 (protein accession number: At3g24300) was amplified from a cDNA pool of *Arabidopsis thaliana* (Col-0) by high-fidelity PCR using the following primers (5′-3′): GTCGAATTC ATGTCAGGAGCAATAACATGC and GTCTCTAGATTAA ACGCGAGGAGGAGTAGC. After verification by sequencing, the ORF of AtAMT1;3 was cloned into pCI vector for oocyte expression as previously described (Hao et al., 2016). Point mutations (see the relevant figures for the positions) were introduced by overlap extension PCR and the mutated sequences were transferred into pCI.

### Electrophysiological Measurements in Oocytes

The care and use of *Xenopus laevis* frogs were reviewed and approved by Laboratory Animal Resources, Chinese Academy of Sciences. Handling of frogs was conducted according to the standard biosecurity and institutional safety procedures. Oocytes preparation, injection and electrophysiological recordings were performed as previously described (Hao et al., 2016). Briefly, oocytes were removed from female *Xenopus laevis* via surgery, then were defolliculated with 1 mg/mL collagenase A (Roche) in Ca^2^^+^-free solution (in mM: 82.5 NaCl, 2 KCl, 1 MgCl_2_, 5 HEPES, pH 7.4) for 1.5 h. The dispersed oocytes were washed 6-8 times with ND96 solution (in mM: 96 NaCl, 2 KCl, 1 MgCl_2_, 1.8 CaCl_2_, 5 HEPES, pH 7.4). Finally, healthy oocytes were selected for micro-injection and kept in ND96 solution. Each oocyte received 60 ng of plasmid *AtAMT1;3*-pCI, *OsAMT1;3*-pCI, or of their mutated forms, using a Nano-liter 2000 micro-injector (WPI, Sarasota, FL, United States). The oocytes were then incubated for 3–4 days in ND96 solution at 22°C. Control oocytes were injected with same volumes of pure water. The currents were recorded by two-electrode voltage-clamp technique supported by the AxoClamp 900A amplifier (Molecular Devices, San Jose, CA, United States). The basal recording solution contained (in mM): 100 NaCl, 2 CaCl_2_, 2 MgCl_2_, 4 Tris, the pH being adjusted to 7.4 with MES. For pH 5.4 and 6.4, 4 mM MES was added and Tris was used to adjust the pH. NH_4_^+^ or MeA^+^ was added (as chloride salt) into the solution as indicated. To allow onsite assessment of current recording quality and reduce the impact of artefactual current instability and fluctuations during the capture of relatively small changes in current intensity, we adopted a stable current recording protocol modified from previous work (Lu et al., 2009; Hao et al., 2016). Briefly, a 1.5 s voltage ramp from − 160 to + 20 mV with extended holding epochs at −70 mV was run continuously, allowing readily visible recordings of the “U” shaped current traces upon the successive introduction and withdrawal of the NH_4_^+^ or MeA^+^ substrate. By the meantime, the peak currents in response to the successively applied voltage ramps were captured and compared. This recording strategy provided reversible current measurements at ramped voltages under strict baseline stability control. NH_4_^+^ or MeA^+^-elicited currents were calculated by subtraction of background “current” recorded in absence of NH_4_^+^ or MeA^+^ from total current recorded in presence of either substrate. Similar to previous reports (Ludewig et al., 2002; Yang S. et al., 2015), oocytes injected with H_2_O (control oocytes) did not produce significant endogenous currents in 1 mM NH_4_^+^ or 10 mM MeA^+^ (and below these concentrations).

The Michaelis-Menten parameters describing the kinetics of NH_4_^+^ and MeA^+^ uptake currents were obtained by fitting the experimental curves with the equation: *I* = *I*_max/_(1 + *K*_m_/c), where *I*_max_ is the maximal current at saturating concentration, *K*_m_ is the substrate concentration corresponding to half-maximal currents, and c is the substrate concentration. The experimental curve describing the voltage dependence of *K*_m_ was fitted using the following equation: *K*_m_ = *K*_m_^(0*mV*)^ × exp (δ × e × V/kT), where δ is fractional electrical distance, V is membrane potential, and e, k and T have their classical meaning, ionic charge, Boltzmann’s constant, and absolute temperature, respectively.

### Sequence Alignments, Homologous Modeling and Molecular Docking

The full-length amino acid sequences of AtAMT1;3 (protein accession number: At3g24300) and EcAmtB (protein accession number: NP 286193) were aligned using DNAMAN 6.0. Transmembrane domains (TMs) and conserved amino acid residues potentially involved in substrate binding and pore-confining were highlighted according to the crystallized EcAmtB protein (Khademi et al., 2004). Sequence comparison among plant AMTs was also focused within the short regions where point mutations were analyzed.

Homology modeling was carried out in collaboration with Beijing Computing Center (Beijing, China) using the crystallized EcAmtB (1U7G) protein (Khademi et al., 2004) as the template by Modeler (Version 4.0) software. Molecular-docking simulation was performed for the prediction of substrate binding using the AutoDock Vina software (version 4.2).

### Data Analysis

Stable recordings of electrophysiological measurements were collected from 3 independent experiments. Data were presented as means ± SE from at least 3 oocytes (in most cases, *n* > 4) from different frogs. Statistical results were obtained using the one-way ANOVA and Student’s *t*-test with SPSS 20.0 software.

## Results

### Establishment of the Stable Recording Strategy for AtAMT1;3

**FIGURE 1:**
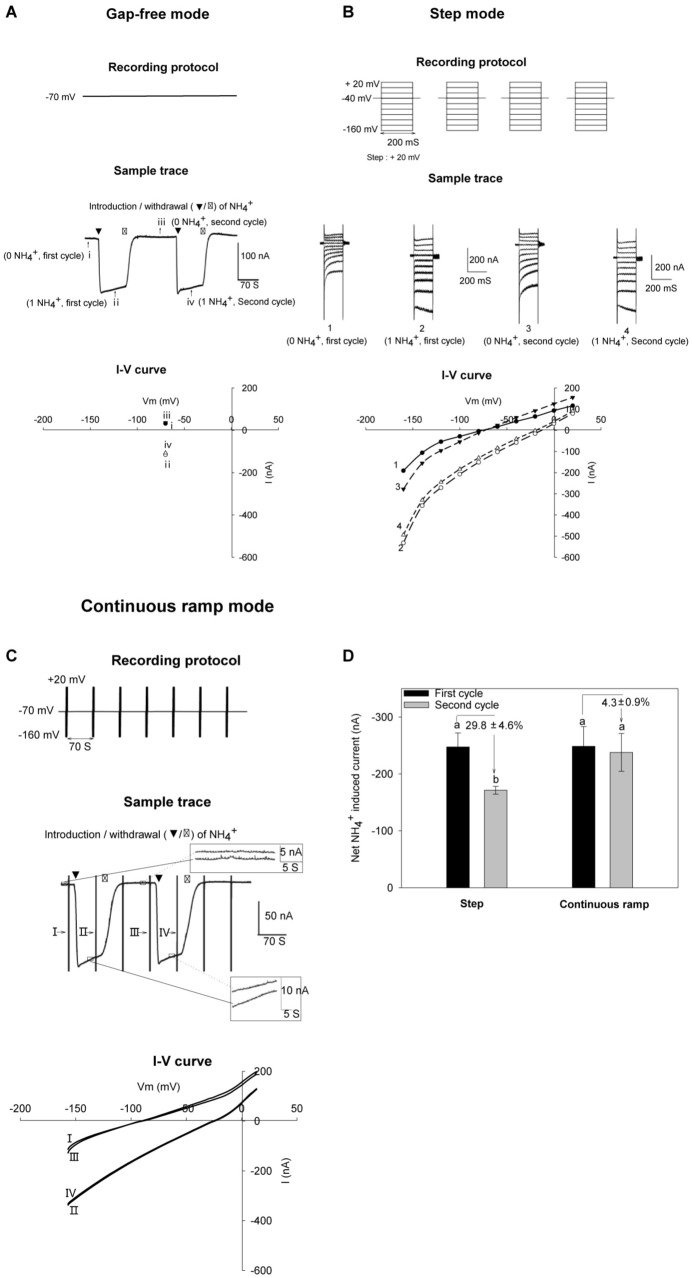
NH_4_^+^ currents recorded with different recording strategies in oocytes. AtAMT1;3-expressing oocytes were subjected to 2 successive cycles of 2 perfusion solutions, the first solution being free from NH_4_^+^ and the second one containing 1 mM NH_4_^+^ (same solution background, pH 7.4). For each cycle, the oocyte was successively bathed in background solution, then NH_4_^+^ solution and then background solution again. Same oocytes were analyzed by different recording strategies. **(A)** Gap-free recordings showing reversible NH_4_^+^-elicited current at – 70 mV. The currents recorded during the first cycle of perfusion with the NH_4_^+^-free background and the 1 mM NH_4_^+^ solutions are indicated by roman numeral i or ii, respectively, and the currents recorded during the second cycle of perfusion with the NH_4_^+^-free and 1 mM NH_4_^+^ solution are indicated by roman numeral iii or iv. **(B)** Strategy for recording NH_4_^+^-elicited currents in response to voltage step increments. NH_4_^+^ current was determined by subtracting the total current recorded in absence of NH_4_Cl (0 NH_4_^+^) from the total current in presence of 1 mM NH_4_Cl (1 NH_4_^+^). Two successive current recordings in the same conditions (during the first and second perfusion cycles) provide an example of possible fluctuations during the progress of recordings. Currents recorded during the first cycle perfusion with the NH_4_^+^-free and 1 mM NH_4_^+^ solutions are indicated by arabic numerals 1 or 2, and the currents recorded during the second cycle of perfusion with the NH_4_^+^-free and 1 mM NH_4_^+^ solutions are indicated by arabic numerals 3 or 4. **(C)** Recording strategy used in this report: the continuous ramp recording protocol. Oocytes were voltage-clamped at – 70 mV except for a 1.5 s epoch, occurring every 70 s and during which a voltage ramp was applied from – 160 mV to + 20 mV (top panel). Vertical bars correspond to the changes in current intensity due to the voltage ramps that were applied during 1.5 s every 70 s (middle panel). The current traces in the insets correspond to magnifications of the boxed steady state currents recorded in the absence of NH_4_^+^ (the two traces displayed in the upper inset) or in the presence of NH_4_^+^ (lower inset). Introduction/withdrawal (▼/▽) of 1 mM NH_4_^+^ is indicated. Current-voltage (I–V) relationship before (traces I and III) and after NH_4_^+^ addition (traces II and IV) obtained from voltage ramps performed during the recording displayed in arrow pointed vertical bars. **(D)** Comparison of the data (NH_4_^+^ induced current intensity) obtained using the voltage step protocol described in panel **(B)** and the continuous ramp recording protocol described in Panel **(C)**. Statistics (*n* = 3) showing that variations between data obtained during the first cycle of solution and the second one can be smaller when using the continuous ramp recording protocol (ca. 5% variation) than when using the classical voltage step protocol (ca. 29% variation). Net NH_4_^+^-induced current were obtained by subtracting the total current recorded in absence of NH_4_^+^ from the current recorded in presence of NH_4_^+^.

Transporters often display low rates of transport of their substrates, which is likely to result in weak currents across the membrane, when compared with classical ion channels. This is the case of AMT transporters when expressed in *Xenopus* oocytes (Ludewig et al., 2002, 2003; Ortiz-Ramirez et al., 2011; Straub et al., 2014; Yang S. et al., 2015; McDonald and Ward, 2016). We compared 3 different methodologies to record and analyze the activity of such systems mediating relatively weak currents, and the variations of these currents upon changes in external solutions and responses to various treatments. First, using the so called ‘gap-free’ recording strategy, the oocytes were continuously voltage-clamped to a fixed membrane potential (e.g., − 70 mV, Figure 1A top panel). As illustrated in Figure 1A middle panel, a background current (baseline) was recorded when the AtAMT1;3-expressing oocyte was bathed in NH_4_^+^-free background solution (pH 7.4). Upon introduction of NH_4_^+^ into the external solution, an inward current was rapidly activated in the oocyte and reached a quasi-plateau. Upon withdrawal of NH_4_^+^ from the external solution, the current quickly returned to the baseline level (Figure 1A middle panel). The occurrence of such ‘U-shaped’ current variations specifically responded to the presence of NH_4_^+^ in the bath solution, providing evidence that AtAMT1;3 mediated NH_4_^+^ uptake. This recording strategy allows to observe the reversibility of the currents under strict onsite stability control, but at a single membrane potential (Figure 1A bottom panel), thus sacrificing useful information that can be obtained from the analysis of the voltage dependency of the current. The second current recording strategy we compared used a classical voltage step protocol, a discrete series of potentials ranging from − 160 mV to + 20 mV with an increment of + 20 mV being successively applied to the oocyte membrane (Figure 1B, top panel). The same oocyte was submitted to 2 successive cycles of perfusion solutions. Using the same solutions as in (A), in each cycle were successively perfused the NH_4_^+^-free background solution, then the NH_4_^+^ supplemented (1 mM) solution and thereafter again the NH_4_^+^-free background solution. The protocol of voltage-clamp was successively applied while the oocyte was bathed in the NH_4_^+^-free solution of the first cycle, the 1 mM NH_4_^+^ of the first cycle, the NH_4_^+^ free solution of the second cycle, and finally the 1 mM NH_4_^+^ of the second cycle. The corresponding current traces are displayed in the middle panel of Figure 1B. For each perfusion cycle, the NH_4_^+^-elicited currents were calculated by subtracting the background current recorded in absence of NH_4_^+^ from the total current recorded in presence of NH_4_^+^ (Lin et al., 2008; Ortiz-Ramirez et al., 2011). Obviously, this recording protocol lacked onsite stability control and could occasionally introduce significant current fluctuations along the recording progress, which could compromise the reliability of the data (Figure 1B, bottom panel).

In order to avoid the imperfections inherent to each of the above 2 recording strategies, and take the advantages of each of them, we established a ‘continuous ramp’ protocol that allowed both onsite stability control of the elicitation of the “U-shaped” currents upon the introduction/withdrawal of the substrate and efficient analysis of the voltage dependency of the current. The recording protocol was modified from our previous work on the rice OsAMT1;3 (Hao et al., 2016). A voltage ramp ranging from − 160 to + 20 mV in 1.5 s was repeatedly run, every 70 s during the whole progress of oocyte recording to capture the current-voltage relationship before, during and after substrate perfusion. Meanwhile, the extended epochs at constant voltage enabled onsite observation of the “U-shaped” currents (also called “washout”) at − 70 mV. The frequency of the continuously applied voltage ramps was determined by the holding epoch duration allowing synchronization of a complete solution change (∼70 s) through the perfusion system. The original recordings displayed a series of “U-shaped” current traces, depending on the number of ramps applied. The U-shaped traces were displayed continuously all along the time course of the recording for analysis (Figure 1C middle panel). A stable recording was defined as satisfying the following 2 criteria: (i) baseline (detected at the holding potential of − 70 mV) fluctuations (top right inset, Figure 1C middle panel) had to be smaller than 5 nA, approaching the detection limit of the instrument (Figure 1C), and (ii) variations between repeatedly captured peak currents (below right inset in Figure 1C middle panel) had to be smaller than 10 nA or within 5–10% of maximal current amplitudes (Figure 1C bottom panel). For accurate measurements of changes in current intensity as small as 20 nA, e.g., at the lowest tested concentrations of substrates (as shown below, Figures 3A,B), such strategy was found to be necessary to get reproducible recordings and reliable data. The net NH_4_^+^-induced currents were calculated by subtracting currents recorded in the NH_4_^+^ free solution from those recorded in the following NH_4_^+^ solution. The advantage of this continuous ramp protocol, in terms of reproducibility of the data, when compared with the data we obtained using the voltage step protocol, is highlighted by Figure 1D. Supplementary Figure 1 further highlights that a dedicated recording strategy was strongly useful to the present study since some differences in current intensities, for instance in response to changes in the concentration of the transported substrate in the external medium, appeared to be not much larger than the instrumental detection limit in these conditions.

### AtAMT1;3 Selectivity for NH_4_^+^

**FIGURE 2:**
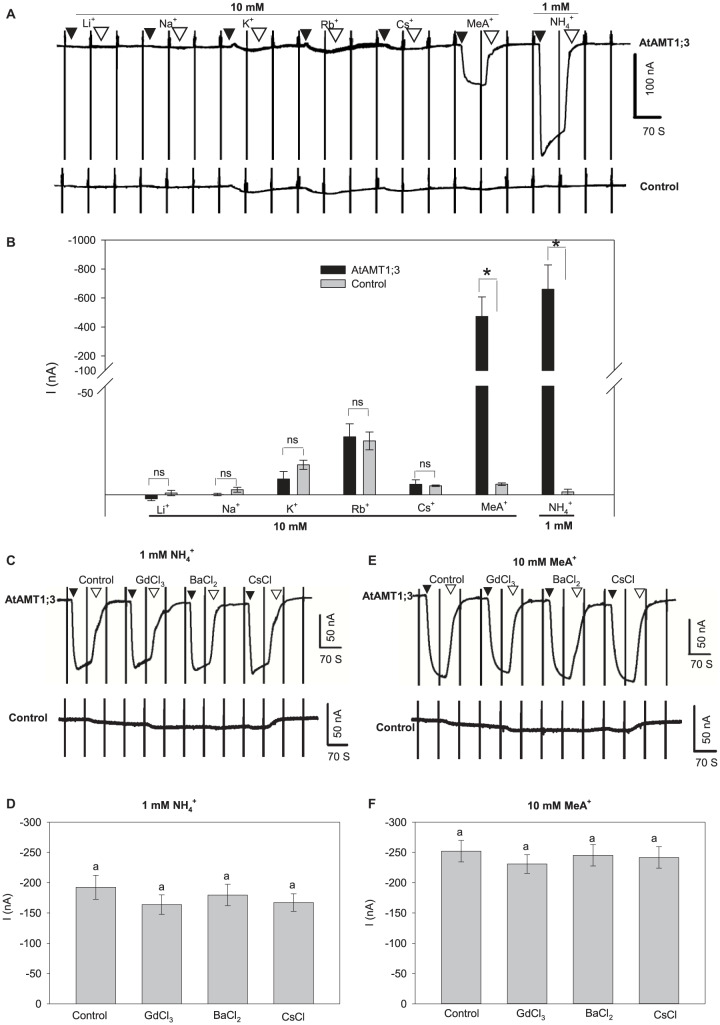
AtAMT1;3 is highly selective for NH_4_^+^. **(A)** Representative recordings of inward currents activated by 10 mM of one of the following monovalent cations, Li^+^, Na^+^, K^+^, Rb^+^, Cs^+^, or MeA^+^ (brought as chloride salts) or by 1 mM NH_4_^+^ (chloride salt) in an *AtAMT1;3*-pCI plasmid injected (upper panel) oocyte or in a control (H_2_O-injected, lower panel) oocyte. Recording protocol: see Figure 1. **(B)** Amplitudes of activated currents determined in AtAMT1;3 expressing or control (H_2_O-injected) oocytes at – 140 mV by addition of 10 mM of either Li^+^, Na^+^, K^+^, Rb^+^, Cs^+^, or MeA^+^, or of 1 mM NH_4_^+^. Means ± SE (*n* = 3). **(C)** Representative recordings of inward currents activated by 1 mM NH_4_^+^ in presence of either 0.1 mM GdCl_3_, 5 mM BaCl_2_ or 1 mM CsCl in an AtAMT1;3 expressing (upper panel) or a control (H_2_O-injected, lower panel) oocyte. **(D)** Amplitudes of activated currents determined in AtAMT1;3 expressing oocytes at – 140 mV by addition to the bath solution of 1 mM NH_4_^+^ only (control) or together with either 0.1 mM GdCl_3_, 5 mM BaCl_2_ or 1 mM CsCl. Means ± SE (*n* = 5). **(E)** Representative recordings of inward currents activated in the presence of 10 mM MeA^+^ and of either GdCl_3_, BaCl_2_ or CsCl (channel inhibitor concentration as in panels **C** and **D**) in an AtAMT1;3 expressing (upper panel) or a control (H_2_O-injected, lower panel) oocyte. **(F)** Amplitudes of activated currents determined in AtAMT1;3 expressing oocytes at – 140 mV by addition of 10 mM MeA^+^ only (control) or together with either GdCl_3_, BaCl_2_ or CsCl (concentrations as in panel **D**). Means ± SE (*n* = 4). Significant differences (LSD, *p* < 0.05) between treatments are indicated by “*” or different letters; not statistically significant differences are indicated by “ns” or by the presence of the same letter above the corresponding bars. Introduction/withdrawal (▼/▽) of NH_4_^+^ or MeA^+^ is indicated. No significant endogeneous NH_4_^+^ or MeA^+^ current was elicited in control oocytes injected with water (bottom panels in **A**, **C**, and **E**).

AtAMT1;3-expressing oocytes displaying large substrate-induced currents were selected to investigate the transporter ionic selectivity by comparing the magnitude of the currents recorded in presence of different monovalent cations. Significant inward current could be reversibly recorded upon the perfusion of 1 mM NH_4_^+^ in AtAMT1;3-expressing oocytes but not in H_2_O-injected control oocytes from the same oocyte batch (Figures 2A,B), indicating that AtAMT1;3 is permeable to NH_4_^+^. Methylammonium (MeA^+^) is a widely used analog of NH_4_^+^. Addition of MeA^+^ at a ten-fold higher concentration (10 mM) than that of NH_4_^+^ in the previous experiments led to a significant increase in current magnitude, similar to that induced by the previous addition of 1 mM NH_4_^+^ in AtAMT1;3-expressing oocytes. Since the control oocytes did not display any significant response to this 10 mM MeA^+^ treatment (Figures 2A,B), the conclusion was that AtAMT1;3 can also mediate MeA^+^ transport. Addition into the perfusion solution of other monovalent cations, Li^+^, Na^+^, K^+^, Rb^+^, or Cs^+^, at 10 mM like for MeA^+^, resulted in similar tiny currents in both AtAMT1;3-expressing and control oocytes (Figures 2A,B), indicating that AtAMT1;3 is not permeable to these monovalent cations. Therefore, AtAMT1;3 is highly selective for NH_4_^+^ and its analog MeA^+^ against other ions. The currents induced by 1 mM NH_4_^+^ and 10 mM MeA^+^ in AtAMT1;3-expressing oocytes were resistant to Gd^3+^, an inhibitor of non-selective cation channels (Burckhardt and Frömter, 1992) and to Ba^2+^ and Cs^+^, which are classical inhibitors of voltage-gated potassium channels (Véry et al., 1995; Su et al., 2005; Yang G. et al., 2015) (Figures 2C–F). No significant endogenous current was activated by either NH_4_^+^ or MeA^+^ in control oocytes, which displayed NH_4_^+/^MeA^+^-induced currents weaker than 10 nA in such conditions (Supplementary Figures 2A,B). Altogether, these results discard the hypothesis that the NH_4_^+^ and MeA^+^ responsive currents recorded in AtAMT1;3-expressing oocytes result from oocyte endogenous cation channel activity that would be artifactually activated by AtAMT1;3 expression or activity. They also provide information on AtAMT1;3 pharmacology, revealing that the permeation pathway of this system is not blocked by any of the classical inhibitors of cation channel activity.

### AtAMT1;3 Binding Affinity for NH_4_^+^ and MeA^+^ Substrates

**FIGURE 3:**
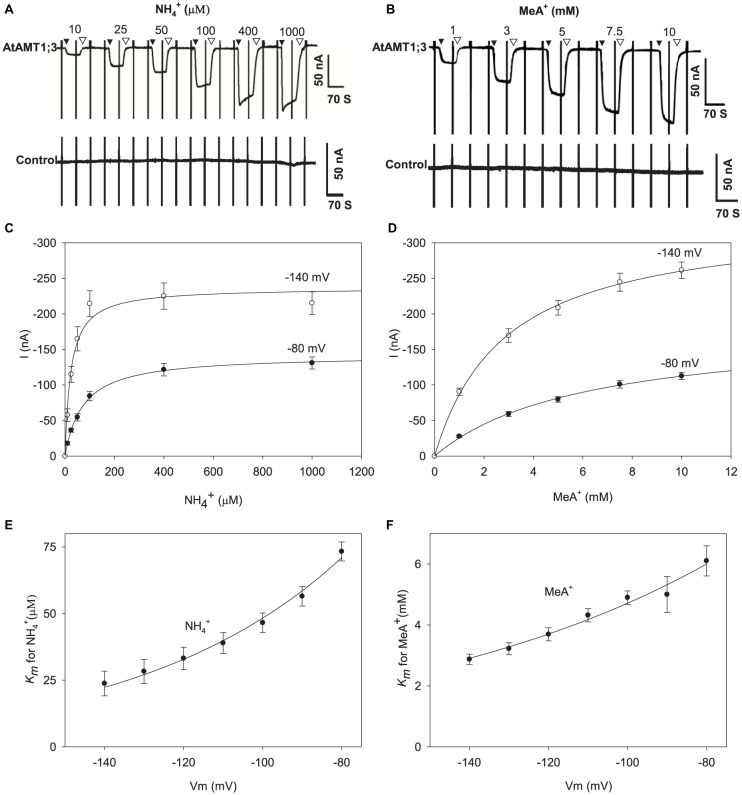
NH_4_^+^ and MeA^+^ transport kinetics in AtAMT1;3 expressing *Xenopus* oocytes. **(A)** Representative recording showing the current response to voltage ramps from – 160 mV to + 20 mV in an AtAMT1;3 expressing (upper panel) or control (H_2_O-injected, lower panel) oocyte bathed in solutions differing in their NH_4_^+^ concentration: 0 (background solution: baseline), 10, 25, 50, 100, 400, and 1000 μM. **(B)** Effect of the external concentration of MeA^+^ on the inward currents recorded in AtAMT1;3 expressing oocytes (upper panel) or control (H_2_O-injected, lower panel) oocytes, investigated as in panels **(A)** for NH_4_^+^. The external concentration of MeA^+^ was fixed to 0 (same background solution as in panel **(A)**, 1, 3, 5, 7.5, and 10 mM. **(C)** and **(D)** NH_4_^+^ **(C)** and MeA^+^ **(D)** transport kinetics for AtAMT1;3 at – 80 mV or – 140 mV. The lines are Michaelis-Menten fits (see section “MATERIALS AND METHODS”). **(E)** and **(F)** Voltage dependence of the *K*_m_ values for NH_4_^+^ **(E)** or MeA^+^ **(F)**. The lines are fits obtained from the exponential function described in the section MATERIALS AND METHODS. Data shown are means ± SE (*n* = 9 for NH_4_^+^; *n* = 6 for MeA^+^). Introduction/withdrawal (▼/▽) of NH_4_^+^ or MeA^+^ is indicated.

AtAMT1;3 permeability to NH_4_^+^ and MeA^+^ was further investigated by analyzing the dependency of the current magnitude on substrate concentration (Figure 3). The NH_4_^+^-induced current amplitude rapidly increased when the concentration of this cation was increased in the μM range and then reached a quasi-plateau at concentrations higher than about 100 μM (Figure 3A and Supplementary Figure 3A). Similarly, the MeA^+^-induced current was sensitive to the concentration of this cation, but saturated at higher concentrations, > 7.5 mM (Figure 3B and Supplementary Figure 3B). No significant endogenous currents (i.e., > 10 nA) were activated by either NH_4_^+^ or MeA^+^ in control oocytes under such conditions (Figures 3A,B, lower panels, Supplementary Figures 2C,D). Further kinetic analysis of the concentration responses of NH_4_^+^ and MeA^+^-induced currents revealed that the uptake of both substrates in AtAMT1;3 could be described by a classical Michaelis-Menten kinetics with the *K*_m_ value in the micromolar and millimolar ranges for NH_4_^+^ and MeA^+^, respectively (Figures 3C,D). At − 80 mV, using the Michaelis-Menten formalism, the *V*_max_ of NH_4_^+^ uptake was − 142 ± 2 nA and this value nearly doubled (− 237 ± 11 nA) at − 140 mV, suggesting a strong facilitation of NH_4_^+^ uptake under hyperpolarization conditions (Figure 3C). The uptake of MeA^+^ was similarly sensitive to membrane hyperpolarization: the *V*_max_ value was − 181 ± 7 nA at − 80 mV and reached − 335 ± 7 nA at − 140 mV (Figure 3D).

Deduced from Michaelis-Menten analysis, the Hill coefficient equals 1, indicating that a single substrate binding event was associated to the transport process. Consistent with the above described sensitivity of the current magnitude to membrane hyperpolarization, reduced *K*_m_ values were observed at more polarized voltages (Figures 3E,F), indicating that the chemical species transported by AtAMT1;3 were in the positively charged ionic form. For instance, at − 80 mV the *K*_m_ values for NH_4_^+^ and MeA^+^ were respectively 73 μM and 6.1 mM, whereas these values decreased to 24 μM and 2.9 mM at − 140 mV (Figures 3E,F). Within this voltage range, the affinity for MeA^+^ was 83–120 folds lower than that for NH_4_^+^, implying mechanistically different bindings for NH_4_^+^ and MeA^+^. Regarding the conclusion of a single substrate binding event in AtAMT1;3, the relative position of the binding sites for NH_4_^+^ and MeA^+^ were assessed (from the voltage dependency of the *K*_m_ parameter; see section “MATERIALS AND METHODS”) to locate at 50% and 31% deep down the membrane electric field, respectively. Thus, from this calculation, MeA^+^ binds to a more superficial position than NH_4_^+^ in the substrate transporting vestibule formed by trimerized AtAMT1;3 proteins, which could explain that the affinity of AMT1;3 is much lower for MeA^+^ than for NH_4_^+^.

### The Uptake of NH_4_^+^ and MeA^+^ Is Independent of Protons

**FIGURE 4:**
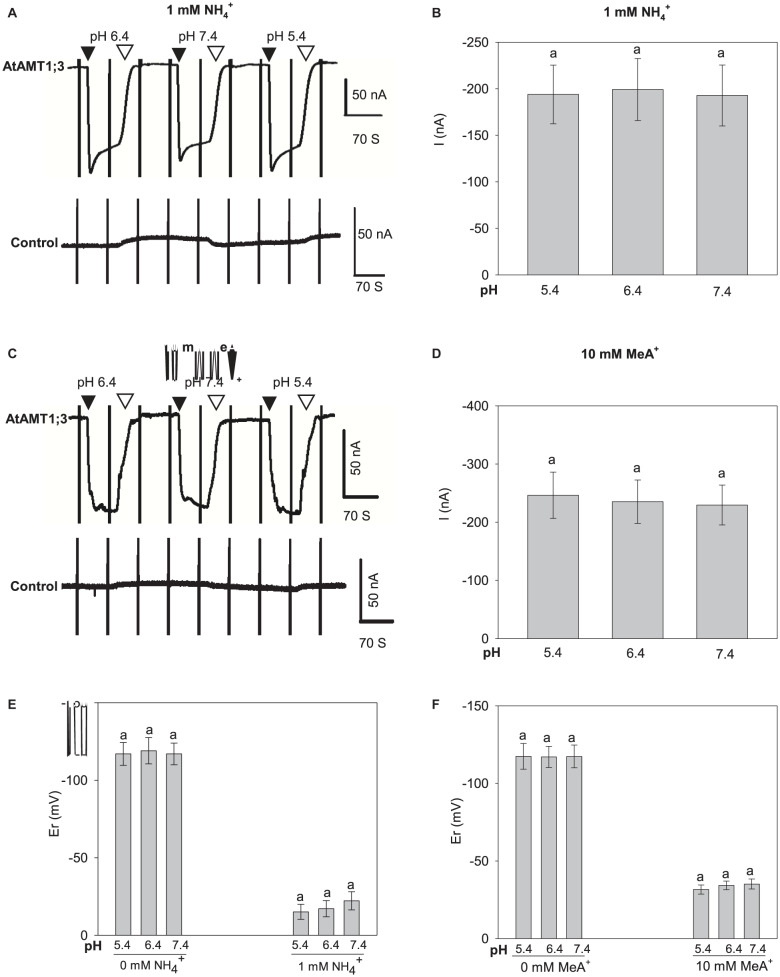
pH dependency of NH_4_^+^ and MeA^+^ induced currents in AtAMT1;3 expressing *Xenopus* oocytes. **(A)** Representative recording showing the current response to 1 mM NH_4_^+^ at different pH values and to voltage ramps from – 160 mV to + 20 mV in an AtAMT1;3-expressing (upper panel) or a control (H_2_O-injected, lower panel) oocyte. The background solution (baseline) was supplemented with 1 mM NH_4_^+^ at pH 5.4, 6.4, or 7.4. **(B)** Amplitudes of activated currents inAtAMT1;3 expressing oocytes at – 140 mV in presence of 1 mM NH_4_^+^ at different pHs as in panel **(A)**. **(C)** and **(D)** Effect of external pH on the activated inward currents recorded in AtAMT1;3 expressing oocytes (upper panel) or control (H_2_O-injected, lower panel) oocytes in presence of 10 mM MeA^+^, investigated as in panels **(A)** and **(B)** for the pH dependency of AtAMT1;3 transport activity in 1 mM NH_4_^+^. **(E)** and **(F)** Effect of external pH on the current reversal potential (Er) in absence or presence of 1 mM NH_4_^+^ **(E)**, or in absence or presence of 10 mM MeA^+^ **(F)**. Means ± SE; *n* = 4 in panels **(B)** and **(E)**, and *n* = 6 in panels **(D)** and **(F)**. Introduction/withdrawal (▼/▽) of 1 mM NH_4_^+^ or 10 mM MeA^+^ at different pH values is indicated.

To check the involvement of H^+^ in the transport of NH_4_^+^ and MeA^+^, we performed current measurements at different external pH values. To obtain sufficiently measurable current amplitudes, 10 mM MeA^+^ was used in these experiments. No significant currents nor current changes were detected in control oocytes, indicating that no endogenous activities were induced by the changes in pH (Figures 4A,C bottom; Supplementary Figures 2E,F). Increasing the external pH from 5.4 to 6.4 and 7.4 had no effect on the currents induced by either 1 mM NH_4_^+^ (Figures 4A,B) or 10 mM MeA^+^ (Figures 4C,D) in AtAMT1;3-expressing oocytes. Assuming that the neutral species of ammonium, NH_3_, was the final form being transported through the pore in a proton coupled manner, greater currents would be measured at lower pH since a pH change from 7.4 to 5.4 would result in a 100-fold increase in proton concentration. The above data however, was not supportive for this assumption. Another possibility may be that protons themselves served as charge carriers and elicited currents. In this case, a pH change would lead to changes in current amplitudes. Furthermore, if protons were transported through the substrate permeation pore, in the absence of NH_4_^+^ or MeA^+^, changes in external pH should lead to shifts in the current reversal potential (Er, the membrane voltage at which no net transport of ions occurs, i.e., no current is observed). However, the Er steadily remained unaffected under different pH with or without the presence of NH_4_^+^ or MeA^+^ (Figures 4E,F), indicating that protons did not play a role in the transport process. Therefore, this set of data clearly ruled out the possibility of the involvement of H^+^ as current carrier. Furthermore, Er shifts toward more positive voltages were immediately observed upon the addition of NH_4_^+^ or MeA^+^ in the external solution, providing further evidence that they were the substrates electrogenically moved by the AtAMT1;3 protein (Figures 4E,F). Thus, altogether, the facts that: (i) significant inward currents occurred specifically in response to the presence of NH_4_^+^ or MeA^+^, (ii) a transport mechanism involving a single substrate-binding event was deduced from the Michaelis-Menten analysis, and (iii) protons were not involved in the transport process, support the hypothesis that AtAMT1;3 functions as an NH_4_^+^ uniporter.

### Regulation of the Transport Activity of AtAMT1;3

**FIGURE 5:**
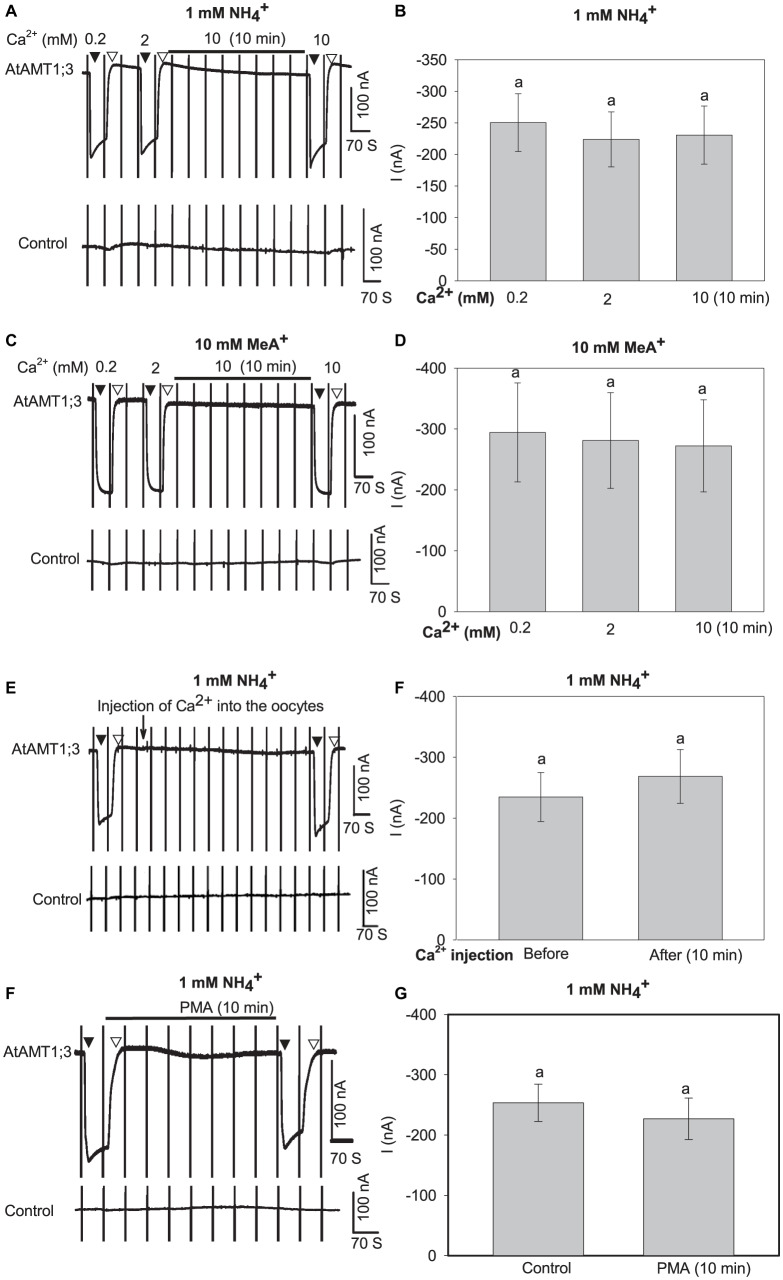
Effects of Ca^2+^ and PMA on AtAMT1;3 transport activity in *Xenopus* oocytes. **(A)** Representative current recording showing the response of an AtAMT1;3-expressing (upper panel) or a control (H_2_O-injected, lower panel) oocyte to 1 mM NH_4_^+^ in presence of either 0.2 or 2 mM Ca^2+^ in the background solution, or in presence of 10 mM Ca^2+^ after 10 min perfusion with the background solution added with 10 mM Ca^2+^. Voltage ramps from – 160 mV to + 20 mV were applied every 70 s as described in Figure 1. **(B)** Amplitudes of activated currents in AtAMT1;3 expressing oocytes at – 140 mV in presence of 1 mM NH_4_^+^ at different Ca^2+^ concentrations introduced in the perfusion solution as described in panel **(A)**. **(C)** and **(D)** Investigation of the effect of Ca^2+^ on AtAMT1;3 transport activity in presence of 10 mM MeA^+^. Same protocol as in panels **(A)** and **(B)** except that 1 mM NH_4_^+^ was replaced by 10 mM MeA^+^. **(E)** Representative current recordings showing the responses of an AtAMT1;3-expressing (upper panel) or a control (H_2_O-injected, lower panel) oocyte to 1 mM NH_4_^+^ before and after the onsite injection of CaCl_2_ (2 mM, 23 nl, indicated by the arrow). The external solution contained 2 mM Ca^2+^ (usual background solution). **(F)** Amplitudes of activated currents determined (as in panel **E**) in AtAMT1;3 expressing oocytes at – 140 mV in presence of 1 mM NH_4_^+^ before (control) or 10 min after injection of Ca^2+^ into the oocytes. **(G)** Representative current recording in a test of the effect of 100 nM PMA pretreatment on an AtAMT1;3-expressing (upper panel) or a control (H_2_O-injected, lower panel) oocyte in presence of 1 mM NH_4_^+^ (in usual background solution). NH_4_^+^ was introduced into the perfusion solution before or after 10 min pretreatment with 100 nM PMA. **(H)** Current activation amplitudes measured (as in panel **G**) in AtAMT1;3 expressing oocytes at – 140 mV in presence of 1 mM NH_4_^+^ before (control) or after 10 min treatment with 100 nM PMA. Means ± SE, *n* = 5, 4, 6, and 6 in panels **(B)**, **(D)**, **(F)** and **(H)**, respectively. No statistically significant difference (LSD for extracellular Ca^2+^ assays, *p* < 0.05; Student’s *t*-test for Ca^2+^ injection and PMA treatment, *p* < 0.05) appeared between the tested treatments. Introduction/withdrawal (▼/▽) of 1 mM NH_4_^+^ or 10 mM MeA^+^ is indicated.

The above results have demonstrated that protons do not play any significant role in regulation of the uptake activity of AtAMT1;3 (Figure 4). Since numerous studies have shown that the activity of different ion channels and transporters is modulated by external Ca^2+^ or Ca^2+^-activated phosphorylation processes (Becker et al., 2004; Ivashikina et al., 2005; Yang S. et al., 2015), we firstly investigated the effect of Ca^2+^ on AtAMT1;3 transport activity. A 10-fold increase in the external concentration of Ca^2+^ (from 0.2 mM to 2 mM) as well as 10-min pretreatment with 10 mM Ca^2+^ did not result in any significant change in the currents recorded in either AtAMT1;3-expressing or control oocytes in response to 1 mM NH_4_^+^ or 10 mM MeA^+^ (Figures 5A–D and Supplementary Figures 2G,H) oocytes. The same Figures show that 10 min incubation in presence of a high concentration of Ca^2+^ (10 mM), expected to raise the oocyte intracellular Ca^2+^ concentration, was also without any significant effect on NH_4_^+^ and MeA^+^-induced currents in AtAMT1;3-expressing and control oocytes. Total withdrawal of Ca^2+^ from the perfusion solution was also without any effect on the currents (Supplementary Figures 4A–D). Lastly, in presence of 2 mM Ca^2+^ in the external solution as well as in absence of this cation, direct injection of Ca^2+^ (23 nL of 2 mM Ca^2+^, brought as chloride salt) into AtAMT1;3-expressing and control oocytes did not result in any significant change in NH_4_^+^-induced currents (Figures 5E,F and Supplementary Figures 2I, 4E,F). Taken together, these results indicated that AtAMT1;3 is not regulated by Ca^2+^ either extracellularly or intracellularly. Prolonged application of a potent protein kinase C (PKC) activator, phorbol 12-myristate 13-acetate (PMA, for references, see Green et al., 2011; Pryazhnikov et al., 2011; Wang Y. et al., 2016; Zhang et al., 2017), at 100 nM for 10 min was also without any significant effect on the uptake of NH_4_^+^ in AMT1;3-expressing oocytes (Figures 5G,H), like in control oocytes (Supplementary Figure 2J). Therefore, AtAMT1;3 does not appear to be regulated by Ca^2+^-activated PKC mediated phosphorylation processes. Previous analyses have also shown that this protein is not regulated by the Ca^2+^-sensing kinase complex CBL1/CIPK23 (Straub et al., 2017).

### Homology Modeling and Prediction of Substrate Binding

The above functional analyses indicated that AtAMT1;3 clearly differed from most plant AMTs characterized so far with respect to the affinity for MeA^+^ compared to that for NH_4_^+^. In most of the previously characterized AMTs, the MeA^+^ to NH_4_^+^ *K*_m_ ratio [*K*_m_(_MeA+_)/*K*_m_(_NH4+_)] was below 30 (Ludewig et al., 2002; Wood et al., 2006; Neuhäuser et al., 2007; Loqué et al., 2009), while this ratio in AtAMT1;3 can reach values higher than 100 (Figures 3E,F). This extremely high parameter may indicate a special substrate binding mechanism for AtAMT1;3.

**FIGURE 6:**
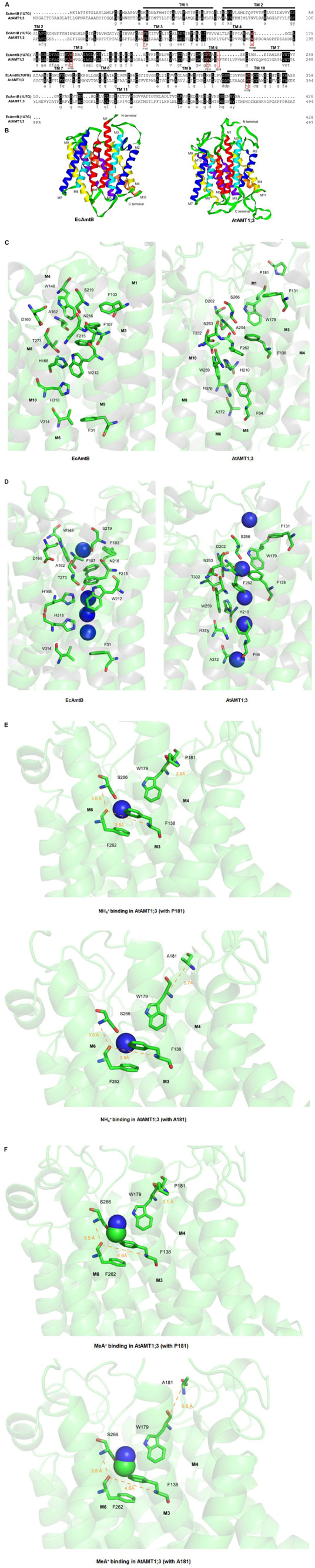
Analysis of homology model of AtAMT1;3. **(A)** Amino acid sequence alignment between AtAMT1;3 and EcAmtB. Substrate transport key residues EcAmtB, F107, W148, H168, F215, S219 and H318, are indicated within red boxes. The numbering of the F107, W148, H168, F215, S219 and H318 in EcAmtB was relative, according to custom, since it was recounted from the 23th amino acid based on the crystal structure study (Khademi et al., 2004). **(B)** Ribbon structure of the crystal structure of EcAmtB (PDB: 1U7G) and the homology model of AtAMT1;3. **(C)** and **(D)** Status of substrate transport key residues in EcAmtB and homology model of AtAMT1;3 in the absence **(C)** or presence **(D)** of NH_4_^+^ ions in the permeation pathway. **(E)** and **(F)**, NH_4_^+^ **(E)** and MeA^+^ **(F)** binding sites in the homology model of AtAMT1;3. Note that for the purpose of a close scenery comparison, the model of AtAMT1;3 (Right panel) was presented by the same visual angle as EcAmtB (Left panel). This introduced an optical illusion that the second bottom NH_4_^+^ ion seems to appear ‘inside’ the benzene ring of F64. However, the predicted position of this NH_4_^+^ ion is behind the ring.

**FIGURE 7:**
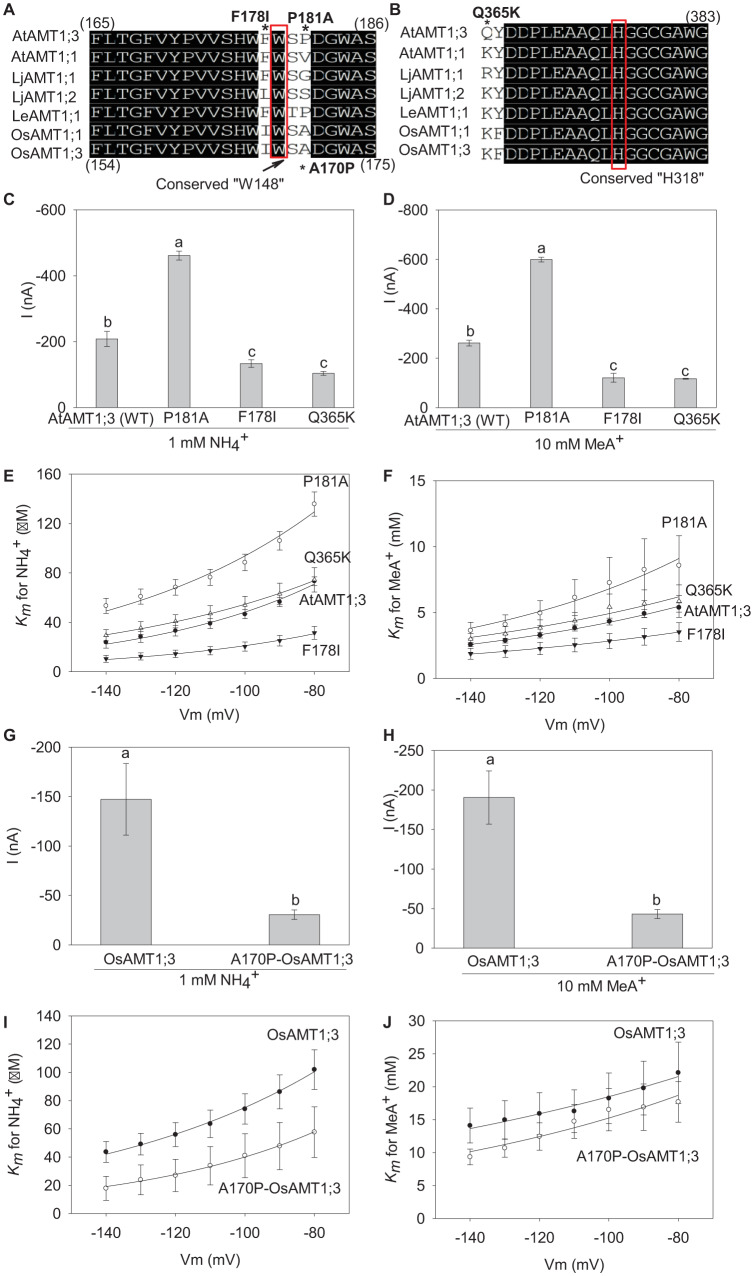
Analysis of the effects of selected point mutations on transporter properties. **(A)** and **(B)** Amino acid sequence alignments of plant AMTs around the key conserved substrate binding residue W148 **(A)** and the second pore-confining H318 residue **(B)** of EcAmtB. In these alignments, the symbol * indicates the positions of the point mutations investigated in the present study: F178I, P181A and Q365K in AtAMT1;3, and A170P (opposite of P181A in AtAMT1;3) in OsAMT1;3. **(C)** and **(D)** Currents induced by 1 mM NH_4_^+^ **(C)** or 10 mM MeA^+^ **(D)** at – 140 mV in oocytes expressing either the P181A mutant, the F178I mutant, the Q365K mutant or the wild-type AtAMT1;3 transporter. **(E)** and **(F)** Voltage dependence of the *K*_m_ for NH_4_^+^ **(E)** or MeA^+^ **(F)** in the P181A mutant, the F178I mutant, the Q365K mutant or the wild-type AtAMT1;3 transporters. Means ± SE. For the NH_4_^+^ data (panels **C** and **E**), *n* = 3 for F178I, *n* = 5 for P181A, *n* = 4 for Q365K, and *n* = 9 for AtAMT1;3. For the MeA^+^ data (panels **D** and **F**), *n* = 4 for F178I, *n* = 3 for P181A, *n* = 3 for Q365K, and *n* = 5 for AtAMT1;3. **(G)** and **(H)** Currents induced by 1 mM NH_4_^+^ **(G)** or 10 mM MeA^+^ **(H)** at – 140 mV in oocytes expressing either the A170P-OsAMT1;3 mutant or the wild-type OsAMT1;3 transporter. **(I)** and **(J)**, Voltage dependence of the *K*_m_ for NH_4_^+^ **(I)** or MeA^+^ **(J)** in the A170P-OsAMT1;3 mutant or the wild-type OsAMT1;3 transporters. Means ± SE: *n* = 3, 3 respectively for the mutant and the wild_type OsAMT1;3 transporter. Significant differences (LSD, *p* < 0.05) between data are indicated by the presence of different letters above the corresponding histogram bars.

At least 60–70% of sequence similarities are typically found among plant AMTs (Couturier et al., 2007). The amino acid sequence alignment indicates that the overall similarity between AtAMT1;3 and EcAmtB is lower, close to 27%, with however relatively higher levels of conservation between transmembrane segments (TMs) (Figure 6A). The low degree of homology with EcAmtB might however indicate that plant AMTs, including AtAMT1;3, functionally differ from their bacterial partner. Nonetheless, key amino acid residues involved in the structure of the central transporting vestibule in EcAmtB are found to be extremely conserved in AtAMT1;3 (Figure 6A), suggesting that the fundamental mode of action resolved with EcAmtB could, to some extent, also apply to AtAMT1;3. This assumption was partly supported by the similar scaffolds predicted for both proteins (Figure 6B). When focused on the putative substrate binding site (S1), the major determinants of the S1 site identified in EcAmtB, W148, S219, F107 and F215 (Figure 6C, left), appear to be strongly conserved in AtAMT1;3, their counterparts being respectively W179, S266, F138 and F262 (Figure 6C, right). But the spatial distribution of these groups is different from that in EcAmtB, and exhibits a more flexible conformation (Figure 6C). Further inside, the deduced pore-lining residues H210 and H376 appear slightly “twisted” in AtAMT1;3 as compared to H168 and H318 in EcAmtB (Figure 6C). These conformational divergences could consequently result in different behaviors between the two proteins concerning substrate binding and passage through the vestibule as predicted under “substrate-free” conditions (Figure 6D). Note that for the purpose of a close scenery comparison, the model of AtAMT1;3 (Right panel) was presented by the same visual angle as EcAmtB (Left panel). This introduced an optical illusion that the second bottom NH_4_^+^ ion seems to appear ‘inside’ the benzene ring of F64. However, the predicted position of this NH_4_^+^ ion is behind the ring. In the presence of NH_4_^+^ in AtAMT1;3, hydrogen bonds are predicted to occur between the ^S266^N-O^*F*262^ atoms (distance 3.0 Å) and the ^*F*138^N-O^*F*262^ atoms (distance 3.8 Å), and the bound NH_4_^+^ ion is stabilized by the S1 site by van der Waals force interactions with W179, F138 and F262 (Figure 6E). In this configuration, the P181 residue of the wild type AtAMT1;3 was predicted to form a hydrogen bond interaction with W179 (distance between the α-C of the 2 residues was 2.9 Å, Figure 6E, Upper). Interestingly, when replaced by a simple and small sized amino acid, A, the deduced distance between the replacing A (A181) and W179 was 5.7 Å, Figure 6E, Lower), indicating that no interaction occurred between them. Regarding the binding of MeA^+^, weaker van der Waals force interactions between MeA^+^ and W179, F138 and F262 are predicted to occur, compromising the two above cited H-bond interactions (3.5 Å and 4.6 Å are then present between ^S266^N-O^*F*262^ and ^*F*138^N-O^*F*262^) (Figure 6F). In this configuration however, the predicted interactions of W179 with P181 as well as the lack of interaction of W179 with the replacing A181 residue in the P181A mutant, occur as described above in the case of NH_4_^+^ binding (Figure 6F, upper and lower panels). As the result of these structural features, MeA^+^ is not perfectly stabilized by the binding site, which dramatically alters the binding affinity of the transporter for this cation. Furthermore, in this model, the P181 residue is predicted to be involved in substrate binding for both NH_4_^+^ and MeA^+^. This hypothesis was experimentally tested by analyzing the effects of P181A and of other point mutations on the transporter activity and affinity for NH_4_^+^ and MeA^+^ (see below and Figure 7).

### Identification of Key Residues Involved in Substrate Binding and Permeation

Since sequence/structural divergence often results in functional consequences in membrane transport proteins, we analyzed the contributions of F178, P181 and Q365 residues to AtAMT1;3 transport activity and affinity. These three residues were targeted in AtAMT1;3 based on their divergence, when this transporter is compared with other plant AMTs (like AtAMT1;1) previously shown to behave as NH_4_^+^ (and MeA^+^) uniporters, thus to display similar transport mechanisms as AtAMT1;3, and characterized in terms of *K*_m_ values for NH_4_^+^ and MeA^+^ (Figures 7A,B). F178 and P181 flank the conserved W179 residue (equivalent to W148 of EcAmtB) in the substrate binding site. Q365 is in the neighborhood of H378 (H318 in EcAmtB), one of the two H residues, with H210, that lined the pore and confined the substrate transport activity in the protein. Sequence alignments show that, while the proline residue (which contains a pyrrolidine ring) occupies the above discussed position 181 in AtAMT1;3, small-sized aliphatic amino acid residues V, A or G are present at the corresponding position in most plant AMTs (Figure 7A). Close to W179 and at a position that is not conserved amongst plant AMTs, F178 was also targeted (Figure 7A). Lastly, while the above discussed position 365 in AtAMT1;3 is occupied by the neutral amino acid Q, positively charged K or R residues are displayed at the corresponding position by other plant AMTs (Figure 7B). We therefore investigated the effects of each of the three point mutations P181A, F178I and Q365K in AtAMT1;3, which are assumed to restore the consensus of classical AMTs at these positions. To check whether the conclusions from such analyses regarding the role of P181 could concern also other AMT transporters, we investigated the effects of the point mutation A170P in the rice OsAMT1;3. This mutation, which introduces a P at the position corresponding to P181 in AtAMT1;3, can thus be considered as the opposite of the P181A mutation introduced in OsAMT1;3 (Figure 7A).

When expressed in oocytes, the P181A mutation in AtAMT1;3 was found to approximately double the amplitude of both NH_4_^+^ and MeA^+^ induced currents, whereas the F178I and Q365K mutations reduced these currents by about 50% (Figures 7C,D). Using the Michaelis-Menten formalism to analyze the kinetics of AtAMT1;3 transport in a physiologically significant range of membrane potentials, − 80 to − 140 mV, revealed that, for both substrates, the P181A mutation resulted in a significant increase in the value of the *K*_m_ parameters. Conversely, the F178I mutation led to decreased values of the *K*_m_ parameters. The Q365K mutation let this parameter rather unchanged, when compared with the corresponding value of the wild type AtAMT1;3 transporter (Figures 7E,F). These results indicated that the P181, F178 and Q365 residues contributed to the structure of the permeation pathway, and thereby to the transport capacity of AtAMT1;3, but probably through different mechanisms. Molecular docking based on the model as shown in Figures 6D–F predicts that the P181A mutation widens the binding pocket, and thereby facilitates the migration of NH_4_^+^ and MeA^+^. In contrast, the Q365K mutation, by introducing a large and positively charged residue close to the pore-lining H376 would “squeeze” and obstruct the pore, and thereby reduce the transporter conductance.

In line with the effect of P181A in AtAMT1;3, the opposite mutation A170P in the rice OsAMT1;3 caused a reduction in both the current amplitude and the *K*_m_ values (Figures 7G–J). Combining the findings in AtAMT1;3 and OsAMT1;3, the residue present at the position corresponding to that of P181 in AtAMT1;3 can be predicted to significantly contribute to substrate binding and permeation. Furthermore, the data provide evidence that the residue corresponding to F178 also affects these properties, but in an opposite way.

## Discussion

Although the ammonium transporter AtAMT1;3 is known to be one of the major contributors to *Arabidopsis* root NH_4_^+^ uptake, as this has been shown by reverse genetics and mutant plant phenotyping (Loqué et al., 2006; Yuan et al., 2007), detailed information on its functional properties, and the mechanisms of substrate binding and transport, is still lacking. Investigation of these properties is the major aim of the present study.

### An Electrophysiological Recording Method With Onsite Stability Control for Functional Analysis of Plant AMTs

Electrophysiological analysis in the oocyte expression system has proved to be a powerful approach for investigating the functional properties, and especially the transport mechanisms, of plant AMT transporters. Since the transport activity of most AMTs results in relatively small current intensities, typically in the range of 100–200 nA in oocytes (Ludewig et al., 2002, 2003; Ortiz-Ramirez et al., 2011; Straub et al., 2014; McDonald and Ward, 2016), and because oocytes can display significant endogenous NH_4_^+^ conductance when exposed to concentrations of NH_4_^+^ higher than 1 mM (Burckhardt and Burckhardt, 1997; Ludewig et al., 2002), a method allowing onsite control of baseline and peak current stability during the whole recording period is highly desirable. Such a method should be especially useful for investigating the effects of point mutations or of changes in external conditions, like increases in substrate concentrations (for kinetics analyses), changes in pH or addition of pharmacological effectors, that often result in variations in current amplitude by no more than a few tens of nA. The oocyte recording method used in the present work on AtAMT1;3 combined the advantages of the commonly used “washout” and “step” recording protocols and overcame the contradictory problems associated with these protocols. Thus, such a method can be predicted to be especially useful for electrophysiological analysis of weak currents, and therefore small-sized responses to changes in environmental conditions, as it is the case with currents carried by plant AMTs and many other electrogenic transporters of solutes such as amino acids, small peptides and sucrose for example. However, this method may be not suited for certain particular purposes, for example for characterizing voltage-gated potassium channels, due to its incapability to allow the recording of channel activation/deactivation kinetics (examples for voltage-gated potassium channels, see Véry et al., 1995; Su et al., 2005; Yang G. et al., 2015; Wang L. et al., 2016).

### AtAMT1;3 Transport Mechanisms

All the plant AMTs belonging to subfamily 1 (AMT1 members) characterized so far have been shown to be able to mediate inward currents, indicating that positively charged substrates are transported through the membrane-spanning pathway (for references, see e.g., Ludewig et al., 2002, 2003; Søgaard et al., 2009; Ortiz-Ramirez et al., 2011; Yang S. et al., 2015; Hao et al., 2016; McDonald and Ward, 2016). However, electroneutral transport has also been evidenced in several members of the AMT2 subfamily, through which the electrically uncharged NH_3_ gas molecule has been proposed to be the transported substrate (Guether et al., 2009; Neuhäuser et al., 2009). With respect to AtAMT1;3, the induction of specific inward currents by addition of NH_4_^+^ into the external solution (Figures 2, 3) provides a first support to the hypothesis that AtAMT1;3 mediates transmembrane passage of ionic rather than neutral substrates. Deduced from the Michaelis-Menten analysis, the transport mechanism of AtAMT1;3 involves a single substrate-binding event (Figures 3C,D). The present data also show that the transport activity of NH_4_^+^ and MeA^+^ is not affected by changes in external pH, and thus in the transmembrane H^+^ gradient (Figures 4A–D), and that protons are not mechanistically involved in the transmembrane movement of the substrates (Figures 4E,F). Therefore, taken together, these data demonstrate that AtAMT1;3 acts as an uniporter of the ionic substrates NH_4_^+^ (and MeA^+^) independent of protons. Such transport mechanism has been proposed for several plant AMTs from the AMT1 subfamily (e.g., Ludewig et al., 2002, 2003; Wood et al., 2006; Loqué et al., 2009; Yang S. et al., 2015). However, AtAMT1;2, one of the three major contributors to NH_4_^+^ uptake in *Arabidopsis* roots, with AtAMT1;1 and AtAMT1;3, has been proposed to act as an NH_3_-H^+^ symporter, displaying a considerably lower affinity (Neuhäuser et al., 2007; Neuhäuser and Ludewig, 2014), with distinct substrate affinity and transport mechanism, when compared to AtAMT1;3. In contrast, AtAMT1;1 functions as a typical high-affinity NH_4_^+^ uniporter insensitive to external protons (Wood et al., 2006; Loqué et al., 2009), similarly to the functional properties of AtAMT1;3 described here. It is also interesting to note that AtAMT2;1, the only AMT2 subfamily protein found in *Arabidopsis* roots, but whose changes in expression levels (knockdown or over-expression) do not result in any significant effect on high affinity NH_4_^+^ uptake in *Arabidopsis* roots (Sohlenkamp et al., 2002; Yuan et al., 2007), has been proposed to function as an NH_3_ transporter endowed with extraordinarily low affinity for methylammonium (Neuhäuser et al., 2009). The divergence in substrate transport mechanism and affinity in these AMTs can be expected to have physiological significance. To meet the nitrogen demand of *Arabidopsis*, these AMTs are thought to be effectively coordinated according to their substrate affinities, transport mechanisms and spatial localization along/within the roots. The high affinity NH_4_^+^ uniport properties evidenced in AtAMT1;3 (Figures 3, 4) can be expected to be coordinated with the expression pattern of this transporter, in outer cell layers of the root tips as well as in rhizodermal and cortical cells of root zones that develop lateral root primordia (Loqué et al., 2006; Lima et al., 2010), to contribute to efficient acquisition of NH_4_^+^ from the soil, in a similar way as AtAMT1;1.

### Determinants of Substrate Binding

Analysis of transport activity in dependency of substrate concentration can often provide useful information for the prediction of the mechanisms of substrate binding. The *K*_m_ parameter reflects the binding affinity of the transporter for the tested substrate. In *Xenopus* oocytes, AtAMT1;3 behaves as a typical high affinity NH_4_^+^ transporter (*K*_m_ values within a few tens of micromolars), an observation quite consistent with the physiological characterization of qko + AtAMT1;3 mutant plants obtained by crossing the wild type *Arabidopsis* with the quadruple mutant *amt1;1-1 amt1;3-1 amt2;1-1 amt1;2-1* line (qko) (Yuan et al., 2007) or findings from other groups (De Michele et al., 2013; Ast et al., 2017). The *K*_m_ values for NH_4_^+^ and MeA^+^ are voltage-dependent, decreasing when the membrane becomes more polarized (Figure 3), which provides further evidence that positively charged substrates are transported through AtAMT1;3. Further analysis of the Michaelis-Menten fits and of the *K*_m_-voltage relationship (Figures 3C–F) leads to the deduction of a single ion binding event (Hill co-efficient of 1) and to the prediction that the stabilized binding sites for NH_4_^+^ and MeA^+^ are distinct, respectively located at a depth of 50% and 31% of the membrane electric field. Thus, MeA^+^ would bind to a more superficial locus of the permeation pathway than NH_4_^+^, which would result in a significantly lower affinity.

In addition, homologous modeling and substrate docking analyses indicate that the binding of NH_4_^+^ is stabilized by a network of interactions involving F138, F262 and W179 (Figure 6E). This structural conformation does not favor the binding of MeA^+^, a handicap that results in reduced stability (Figure 6F). These calculations and modeling predictions explain the experimental observation that MeA^+^ is transported by AtAMT1;3 with a very weak affinity (several millimolar *K*_m_). This analysis of AtAMT1;3 affinity for MeA^+^ is not physiologically relevant, since this compound is just an “artificial” substrate experimentally used for investigating the transport activity of AMTs of various origins, e.g., by ^14^C-MeA uptake experiments (Marini et al., 1997) or electrophysiological recordings (Ludewig et al., 2002; Søgaard et al., 2009; Ortiz-Ramirez et al., 2011), but it probably indicates that the structural conformation of the permeation pathway of AtAMT1;3 results in a strong selectivity for NH_4_^+^. Additionally, this extremely low affinity for MeA^+^ led us to further investigate the mechanisms underlying substrate binding.

In general, the transport of methylammonium through AMTs is considered to involve the same mechanisms as that of ammonium. However, the observation that the mutation Q57H in the *Arabidopsis* AtAMT1;1 and the mutation H211E in the common bean PvAMT1;1 give rise to an increase in NH_4_^+^ transport activity while resulting in a decrease in MeA^+^ transport activity (Loqué et al., 2009; Ortiz-Ramirez et al., 2011), may indicate that structural differences exist between AMT proteins in recognizing and transporting the two highly cognate substrates. It has been shown, in the bacterial ammonium transporter EcAmtB, that W148, S219 and F107 play major roles in the structure of the NH_4_^+^ binding site (S1), and that the twin conserved histidines H168 and H318 mainly constitute the pore-confining midway region (Khademi et al., 2004; Zheng et al., 2004; Javelle et al., 2006). However, mutation studies showed that the single mutations W148A and S219A in the S1 site significantly increased the transport activity of ^14^C-methylamonium, whereas the single F107A mutation in the “Phe gate” or the triple mutation F107A/W148A/S219A let the transport activity of the mutant proteins very similar to that of the wild type protein (Javelle et al., 2008). By contrast, as evaluated by the uptake of ^14^C-methylamonium and the application of the inhibitors imidazole or thallium, which act as competitors at the substrate binding site, the F215A mutation of the Phe gate abolished the transport activity. Altogether, these results indicated that F107, although being part of the NH_4_^+^ binding site, is not essential to methylammonium conduction whereas F215 is absolutely required (Javelle et al., 2008). In this respect, the precise mechanism of substrate binding to the S1 site is still disputative. The opposite effects of mutations P181A in AtAMT1;3 and A170P in OsAMT1;3 as well as of F178I in AtAMT1;3 provide further evidence of the involvement of residues neighboring the conserved W148 in determining substrate binding properties (Figure 7). Thus, the binding of the substrate is not only determined by the three conserved residues, but must at least include contributions from adjacent sites. Although P181 in AtAMT1;3 is not unique among plant AMTs, it differs from the majority of the counterparts, where a simple and relatively small-sized amino acid such as G, A or V is present at the corresponding position (Figure 7A). Being a heterocyclic imino acid, the existence of the pyrrolidine ring at the place of alpha-amino group makes proline more complex than Gly, Ala or Val, in term of heterocyclic ring endowed with electron distribution that can facilitate the interaction with neighboring residues. Our modeling prediction also reveals a tight hydrogen bond interaction between the α-C of P181 and W179 in wild type AtAMT1;3, and that the P181A mutation results in absence of such an interaction with W179 (Figures 6E,F). The effects of the P181-W179 interactions have been experimentally highlighted by the analysis of substrate binding affinities (Figures 7E,F). Accordingly, as a parallel evidence, the reciprocal mutation A170P in the corresponding site of OsAMT1;3 results in opposite effects on the binding affinities (Figures 7I,J). Finally, the effects of the mutation F178I (Figures 7E,F) provide further support to the conclusion that, in addition to the key structure built by the conserved F138, F262, S266 and W179 in AtAMT1;3, the two residues P181 and F178 flanking W179 contribute to tune substrate binding through interactions with W179.

In EcAmtB, it has been shown that all the mutations targeting the twin pore-confining histidines H168 and H318, except H168E, resulted in loss of transport activity, confirming that these H residues are essential for substrate conductance (Javelle et al., 2006). However, equivalent mutations of H168E in plant or fungal AMTs were found to result in distinct effects on the transport activity (Boeckstaens et al., 2008; Ortiz-Ramirez et al., 2011; Hao et al., 2016). It is thus tempting to hypothesize that, neighboring the key substrate passage residues, other residues strongly contributing to the transport activity of AMTs exist. The effect of Q365K on current amplitude in AtAMT1;3 provides direct experimental support to the above hypothesis (Figure 7). Here our results regarding the effects of the point mutations P181A and F178I indicate that amino acids neighboring the key substrate binding residues (F138, F262 and W179), besides the contribution to the binding activity, may also contribute to structural constraints that govern substrate permeation, and thus participate to the tuning of the transport activity (Figure 7).

In summary, the present study describes a highly-suited electrophysiological recording strategy that enables synchronized capture of the current-voltage relationship under onsite stability control. We demonstrate that AtAMT1;3 functions as an NH_4_^+^ uniporter independent of protons and Ca^2+^. The observation of the extremely low affinity for MeA^+^ displayed by AtAMT1;3 raised the question of the nature of the complex mechanisms that underlie its substrate binding and permeation. We provide evidence that P181 and F178 amino acid residues contribute to the tuning of the substrate binding as well as to permeation properties, and that Q365 modulates substrate permeation.

## Data Availability Statement

The datasets generated for this study are available on request to the corresponding author.

## Ethics Statement

The animal study was reviewed and approved by Laboratory Animal Resources, Chinese Academy of Sciences.

## Author Contributions

Y-HS, HS, and D-LH designed the work and wrote the manuscript. D-LH conducted electrophysiological experiments and analyzed the data. S-YY participated to mutagenesis and modeling work. S-XL performed homologous modeling. J-YZ and Y-NH assisted in data analysis. A-AV contributed to the analysis of transport mechanisms.

## Conflict of Interest

The authors declare that the research was conducted in the absence of any commercial or financial relationships that could be construed as a potential conflict of interest.

## Abbreviations

MeA^+^: methylammonium
AMTs: ammonium transporters
PMA: phorbol 12-myristate 13-acetate
PKC: protein kinase C.
