# Supplementary material for: Functional Characterization of the Arabidopsis Ammonium Transporter AtAMT1;3 With the Emphasis on Structural Determinants of Substrate Binding and Permeation Properties
Source: Front Plant Sci. 2020 May 21;11:571. doi: 10.3389/fpls.2020.00571 (PMC7256485; doi:10.3389/fpls.2020.00571)

Supplementary Figure 1. Capture of small changes in current intensity in oocytes upon shifts in substrate concentration using the recording protocol described in Figure 1. A-B, Representative current recordings upon changes in NH_4^+ (**A**) or MeA^+ (**B**) concentrations in AtAMT1;3- or OsAMT1;3-expressing oocytes (upper and lower panels, respectively). Each "U-shaped" change in current intensity within a given recording provides the oocyte response to a cycle of 3 perfusion solutions, composed of the background solution (free from transported substrate and used for the baseline recording"), then the same background solution supplemented with the transported substrate (NH_4^+ or MeA^+ at the indicated concentration), and again the background solution free from substrate. Same background solution (pH 7.4) as in Figure 1. The current rapidly increased and peaked upon addition of the substrate, and then returned to the baseline with withdrawal of the substrate. For AtAMT1;3, the representative current traces shown here are the same as those displayed in Figure 3A-B. **C-D**, Examples of the variation in current intensity caused by shifts in the concentration of NH_4^+ (**C**) or MeA^+ (**D**) in AtAMT1;3 or OsAMT1;3 expressing-oocytes at − 80 mV or − 100 mV. Current peaks were used for the calculation of the current variations between two successive concentrations (indicated below the bars) of the substrate, the value of the current peak observed in the solution containing the lower substrate concentration being subtracted from that observed in the solution containing the higher substrate concentration. Introduction / withdrawal (▼/▽) of NH_4^+ or MeA^+ is indicated. Data shown are Means ± SE from at least 3 oocytes. In the range of low substrate concentrations, the current increase when the concentration of the substrate was raised from 10 µM to 25 µM in the case of NH_4^+, or from 1 mM to 3 mM in the case of MeA^+, was in the 20-30 nA range at − 80 mV and 30-40 nA at − 100 mV, for both AtAMT1;3 and OsAMT1;3. In the range of high (almost saturating) substrate concentrations, *e.g.*, when the concentration of NH_4^+ was increased from 400 to 1000 µM, or that of MeA^+ from 3 to 5 mM, the resulting increases in current intensities

were in the 5-30 nA range in both transporters whatever the membrane potential. Thus, the magnitude of such responses of the transporters, in terms of current intensity, to an increase in substrate concentration is not much larger than the instrumental detection limit in these conditions, *ca.* 10 nA, confirming that a dedicated recording strategy can benefit such analyses.

Supplementary Figure 2. Summary of statistical results from control oocytes injected with water. All the data are displayed using a scale for the y axis similar to those used in the figures and supplementary figures providing the current intensities observed in AtAMT1;3- or OsAMT1;3-expressing oocytes in the corresponding experiments. **(A)** Intensities of activated currents in control (H_2O-injected) oocytes at －140 mV by addition to the bath solution of1 mM NH_4^+ (control) or of both 1 mM NH_4^+ and either 0.1 mM $GdCl_3$, 5 mM $BaCl_2$ or 1 mM CsCl as described in Figure 2C bottom panel. **(B)** Amplitudes of activated currents in control (H_2O-injected) oocytes at －140 mV by addition to the bath solution of 10 mM MeA^+ (control) or of both 10 mM MeA^+ and either 0.1 mM $GdCl_3$, 5 mM $BaCl_2$ or 1 mM CsCl as described in Figure 2E bottom panel. **(C)-(D)** Amplitudes of activated currents in control oocytes at －140 mV by addition of different concentrations of NH_4^+ **(C)** and MeA^+ **(D)** as described in Figure 3A and Figure 3B bottom panels, respectively. **(E)-(F)** Amplitudes of activated currents in control oocytes at －140 mV by addition of 1 mM NH_4^+ **(E)** or 10 mM MeA^+ **(F)** at different pHs as described in Figure 4A and Figure 4C bottom panels, respectively. **(G)-(H)** Amplitudes of activated currents in control oocytes at －140 mV by addition of 1 mM NH_4^+ **(G)** or 10 mM MeA^+ **(H)** at different Ca^{2+} concentrations introduced in the perfusion solution as described in Figure 5A and Figure 5C bottom panels, respectively. **(I)** Amplitudes of activated currents in control oocytes at －140 mV by addition of 1 mM NH_4^+ before (control) or 10 min after injection of Ca^{2+} into the oocytes as described in Figure 5E bottom panel. **(J)** Amplitudes of activated currents in control oocytes at －140 mV by addition of 1 mM NH_4^+ before (control) or after 10 min treatment with 100 nM PMA as described in Figure 5G bottom panel. Means ± SE, n = 4, 5, 4, 4, 8, 6, 4, 5, 4, 3 in panels A, B, C, D, E, F, G, H, I, and J, respectively. In all these external solutions and treatments, the NH_4^+ or MeA^+ activated currents in control water-injected oocytes were lower than 10 nA at －140 mV.

Supplementary Figure 3. Effect of the external concentration of NH_4^+ and MeA^+ on AtAMT1;3 transport activity in *Xenopus* oocytes. (A)-(B) Amplitudes of activated currents in AtAMT1;3 expressing oocytes at − 140 mV in presence of different concentrations of NH_4^+ **(A)** or MeA^+ **(B)**. The recording process for the NH_4^+ and MeA^+ induced currents was the same as in Figure 3A and Figure 3B, respectively.

Supplementary Figure 4. Effects of Ca^{2+} on AtAMT1;3 transport activity in *Xenopus* oocytes bathed in Ca^{2+}-free solution. (A) Representative current recording showing the response of an AtAMT1;3-expressing oocyte to 1 mM NH_4^+ in presence of 2 mM Ca^{2+} in the external solution and thereafter in absence of Ca^{2+} (for 3 or 7 min). Voltage ramps from − 160 mV to + 20 mV were applied every 70 s as described in Figure 1C. **(B)** Amplitudes of activated currents in AtAMT1;3 expressing oocytes in 1 mM NH_4^+ at − 140 mV in presence of 2 mM Ca^{2+} in the external solution and thereafter in absence of Ca^{2+} (for 3 or 7 min), as described in Panel A. **(C)-(D)** Effect of Ca^{2+} on AtAMT1;3 transport activity in presence of 10 mM MeA^+. Same protocol as in panels A and B except that 1 mM NH_4^+ was replaced by 10 mM MeA^+. **(E)** Representative current recordings showing the responses of an AtAMT1;3-expressing oocyte to 1 mM NH_4^+ before and after the onsite injection of $CaCl_2$ (23 nl of 2 mM solution, indicated by the arrow). The bath solution was free from Ca^{2+}. **(F)** Amplitudes of activated currents (as in panel E) in AtAMT1;3 expressing oocytes at − 140 mV in presence of 1 mM NH_4^+ before (control) or 7 min after injection of Ca^{2+} into the oocytes. In each histogram, the data are normalized to the intensity of the current observed in the treatment corresponding to the left bar of the histogram. Means ± SE, n = 5, 3, 4 in panels B, D and F, respectively. No statistically significant difference (LSD for extracellular Ca^{2+} assays, $p < 0.05$; Student's t-test for Ca^{2+}

injection, $p < 0.05$) appeared between the tested treatments. Introduction / withdrawal (▼/▽) of 1 mM NH_4^+ or 10 mM MeA^+ is indicated.

Supplementary Figure 1

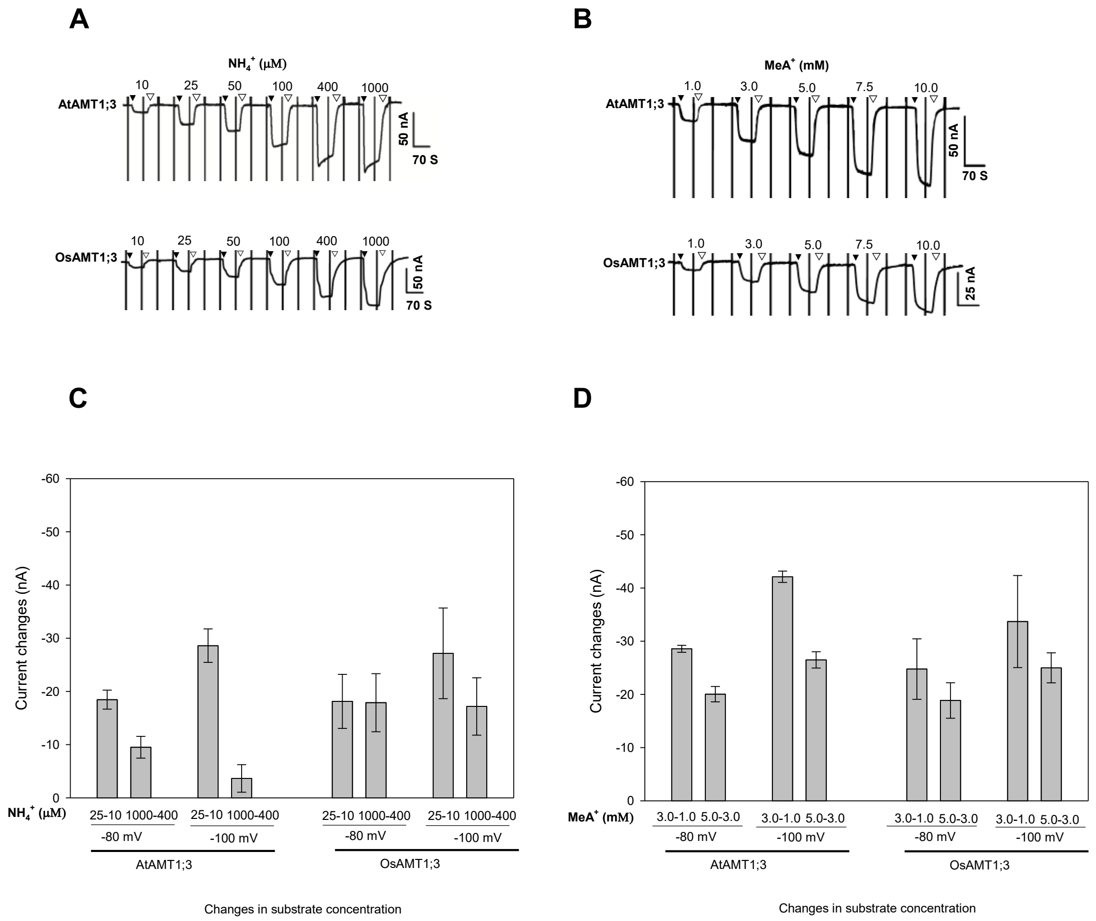

Supplementary Figure 2

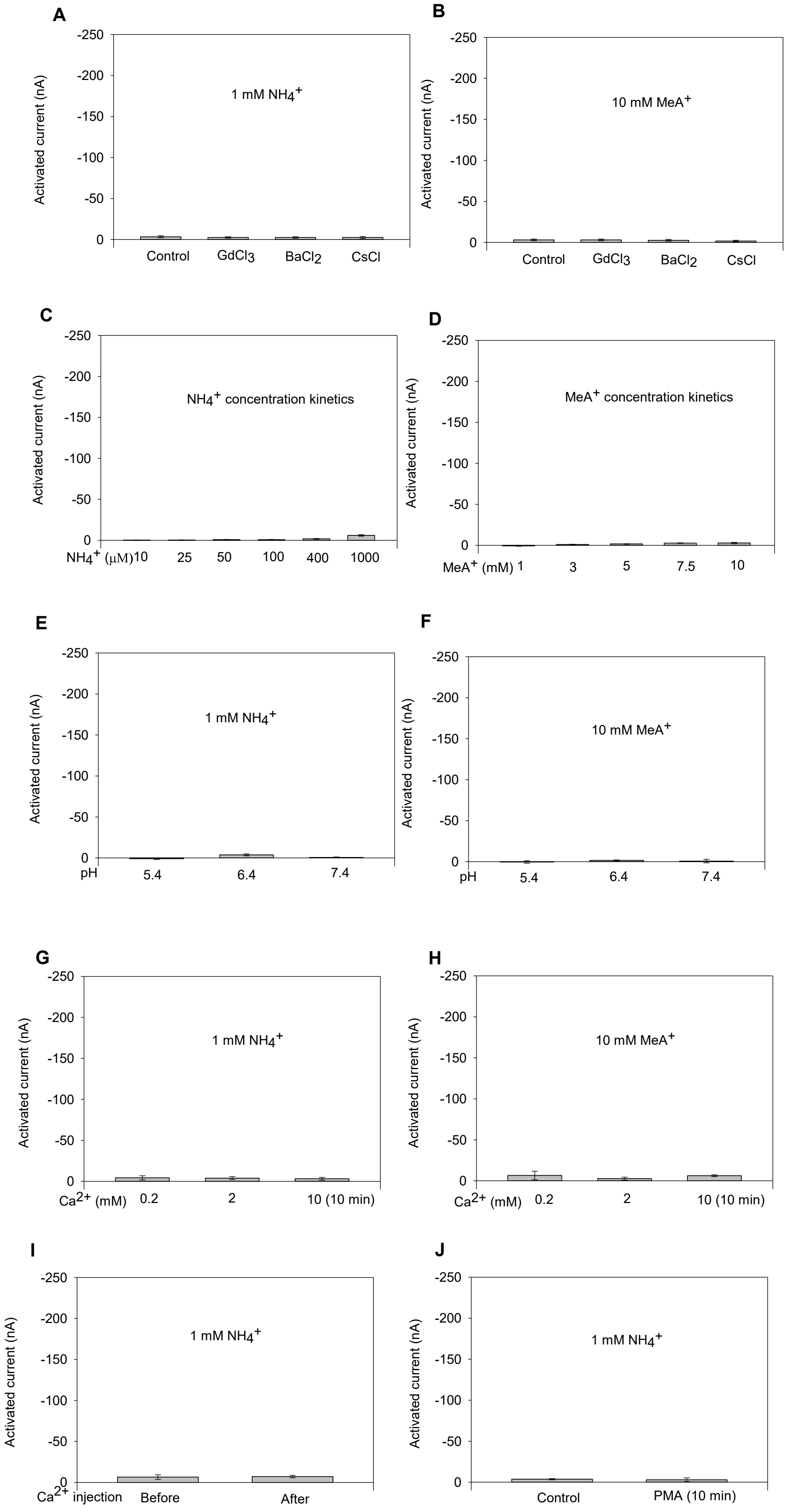

Supplementary Figure 3

A

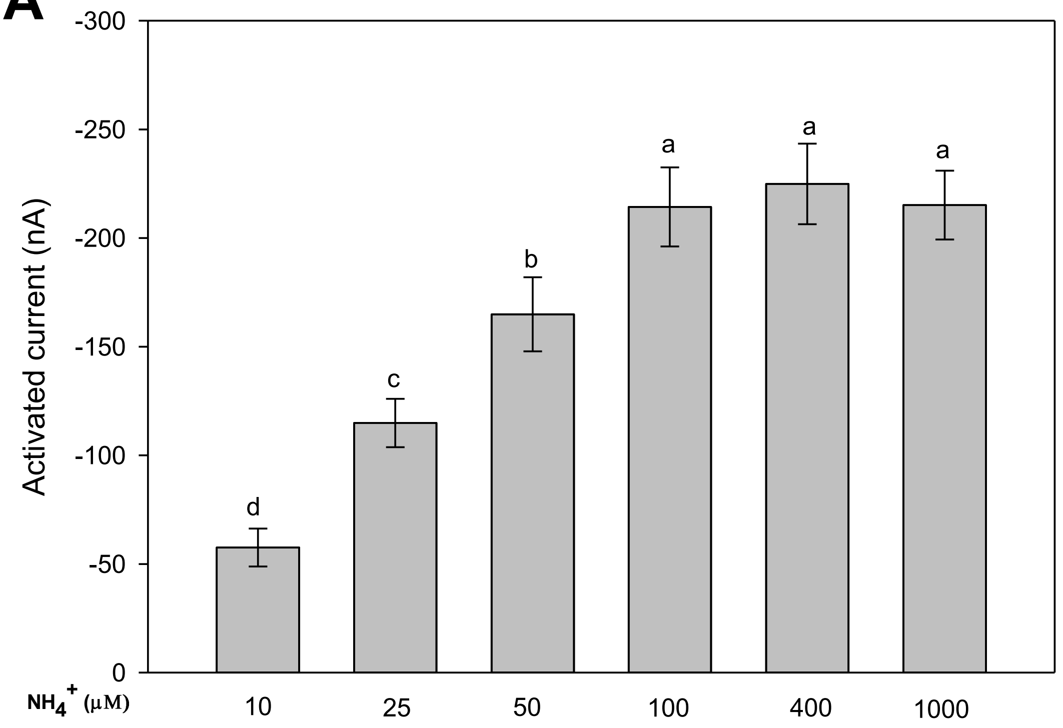

B

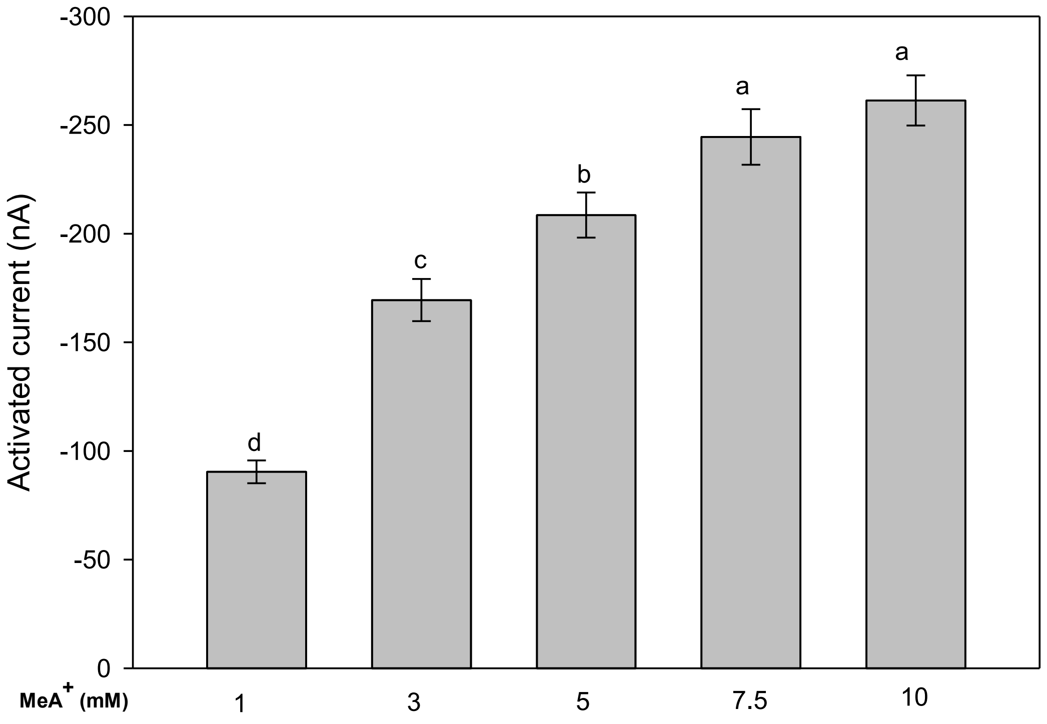

Supplementary Figure 4

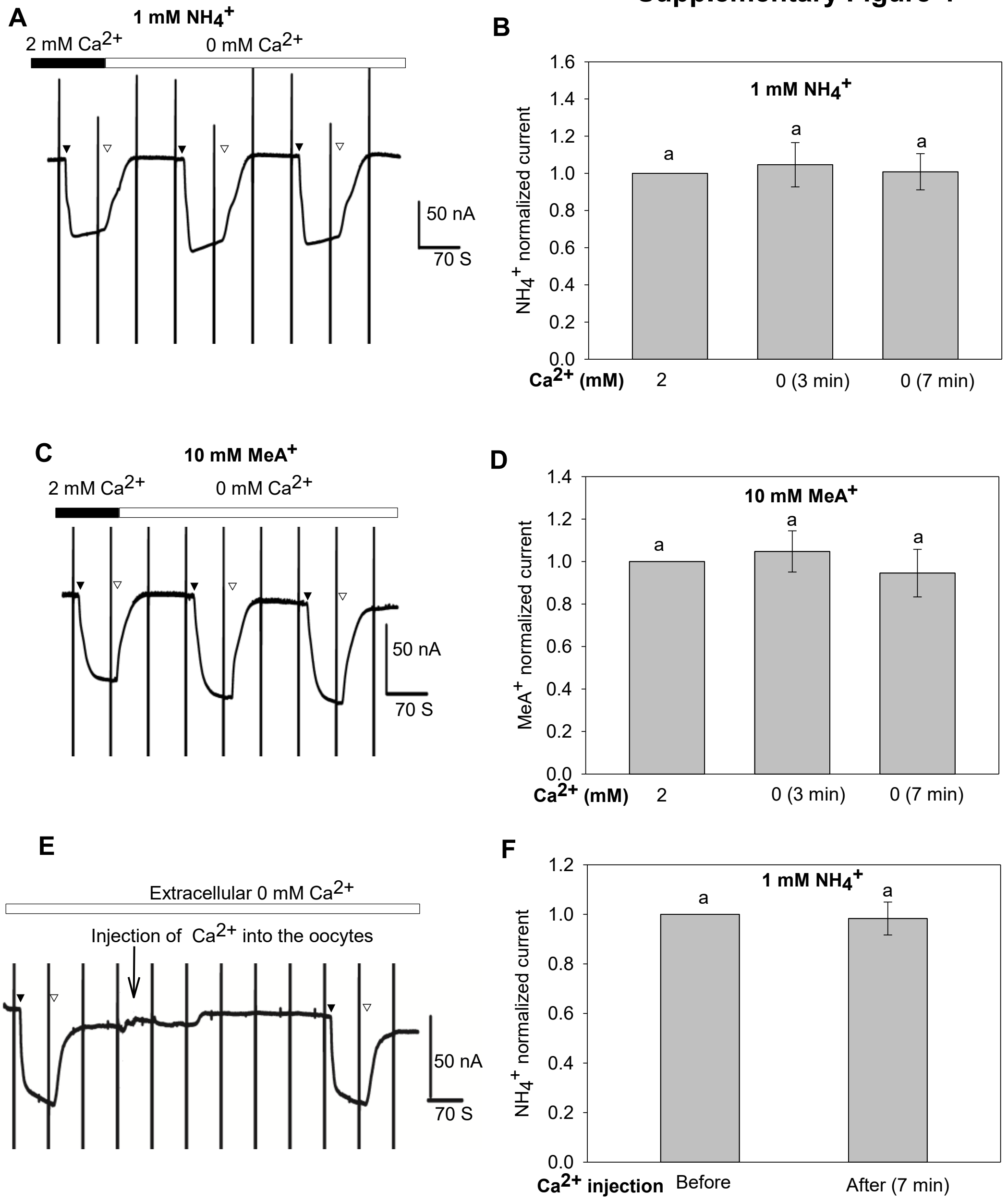

Supplement: Supplementary file 1 [file Presentation_1.pdf]
